# A Haystack Heuristic for Autoimmune Disease Biomarker Discovery Using Next-Gen Immune Repertoire Sequencing Data

**DOI:** 10.1038/s41598-017-04439-5

**Published:** 2017-07-13

**Authors:** Leonard Apeltsin, Shengzhi Wang, H.-Christian von Büdingen, Marina Sirota

**Affiliations:** 1 Department of Neurology, UCSF, San Francisco, 94158 USA; 2 Institute for Computational Health Sciences, UCSF, San Francisco, 94158 USA

## Abstract

Large-scale DNA sequencing of immunological repertoires offers an opportunity for the discovery of novel biomarkers for autoimmune disease. Available bioinformatics techniques however, are not adequately suited for elucidating possible biomarker candidates from within large immunosequencing datasets due to unsatisfactory scalability and sensitivity. Here, we present the Haystack Heuristic, an algorithm customized to computationally extract disease-associated motifs from next-generation-sequenced repertoires by contrasting disease and healthy subjects. This technique employs a local-search graph-theory approach to discover novel motifs in patient data. We apply the Haystack Heuristic to nine million B-cell receptor sequences obtained from nearly 100 individuals in order to elucidate a new motif that is significantly associated with multiple sclerosis. Our results demonstrate the effectiveness of the Haystack Heuristic in computing possible biomarker candidates from high throughput sequencing data and could be generalized to other datasets.

## Introduction

Autoimmune diseases are a significant source of worldwide chronic illness, disability, and death. Early diagnosis is critical to limiting short-and long-term tissue destruction caused by autoimmunity^1^, and clinically actionable biomarkers based on understanding of fundamental pathological mechanisms allowing for timelier diagnoses or clear disease risk stratification may result in significantly improved outcomes^2^. For example, diagnostic certainty early in the disease has greatly improved the clinical care and outcomes of patients with neuromyelitis optica (NMO). NMO is an inflammatory demyelinating disease whose symptoms overlap with those of multiple sclerosis (MS). Prior to the identification of antibodies against aquaporin-4 (AQP4) being specific for NMO spectrum disorders^3^, distinguishing MS from NMO was mainly based on clinical decision-making^4^, and NMO specific treatment algorithms were not available. The discovery of anti-AQP4 antibodies led to the immediate development of blood-based assays for accurate NMO-IgG detection, allowing for earlier diagnosis and prompt initiation of disease-appropriate therapies^5, 6^.

Here, we were interested in developing a computational approach leveraging deep B-cell immune repertoire sequencing data from blood of patients with MS (and healthy donors) to identify disease-specific features that may, after further validation, be used as biomarkers for the early detection of MS. Multiple Sclerosis is an inflammatory autoimmune disease of the central nervous system that involves CNS demyelination and neuronal damage leading to a wide range of debilitating neurological symptoms^7, 8^. MS affects over 2.5 million people in the US and around the world, and there currently is no cure. Although possible causes of the disease include genetic and environmental factors, the actual cause of MS is currently unknown^9^. MS diagnosis presently rests entirely on clinical and MRI data and may include cerebrospinal fluid (CSF) analyses to test for the presence of clonal immunoglobulins, products of clonally expanded CSF B-cells^10, 11^. Increased B-cell levels within a patient’s CSF indicate that an inflammation process which is consistent with MS diagnosis might be ongoing^12^. Scientific evidence suggests that antigen-specific B-cells play a role in the onset and progression of the disease^13^. Antigen-specificity in turn would be encoded in the antigen-recognizing B cell receptor (BCR), surface expressed immunoglobulins, on a highly individual level. Therefore, certain sets of B-cells may serve as MS biomarkers for disease activity or even prediction. There has been some previous work exploring the B-cell and T-cell immune repertoire in MS, however most of the studies have been limited in sample size^14–16^. In our previous work, we showed that clonally related B-cells are present in the actual site of tissue injury^17^.

Any deep examination of a patient’s B-cell repertoire is complicated by the sheer diversity of the B-cell repertoire. On average, the blood of a human adult may contain an estimated 3–9 million distinct B-cell secreted antibodies^18^. The recent advent of high-throughput sequencing technologies has enabled researchers to sample and study the immune repertoire on a large scale^19–23^. These newly developed techniques can now extricate millions of antibody sequences, aiding in studies of lymphocyte malignancies, infectious disease, and autoimmunity^24–27^. In this work, we apply high-throughput sequencing to isolate and catalogue blood-based B-cell DNA from dozens of MS patients and healthy controls (HCs). We present a computational method to query and analyze these data for the purpose of pinpointing potential B-cell related disease biomarkers.

Currently, no protocol exists for calculating biomarker likelihood among a set of antibody sequences. Implicitly, a sequence-associated biomarker may take the form of an amino-acid pattern that correlates with disease diagnosis, and ideally, that pattern would be found exclusively in disease-afflicted patients. Any such ‘Disease-Only Motif’ (DOM) would make a good potential biomarker candidate. Immunoglobulin sequence datasets uniquely lend themselves to efforts directed at DOM determination, as features separating patients from non-patients might be present but deeply hidden in the vast diversity of the experimental data. However, presently available techniques are inadequate for DOM determination, and motif discovery algorithms used to date suffer from a twofold limitation of constrained scalability since algorithms cannot process large sequence quantities and surplus sensitivity to noise since motif quality decreases with increased sequence count^28^, as they are not built to process millions of sequences as input. Scalability issues arise from the dependence of the algorithms on computationally expensive multiple-alignments, while sensitivity errors are caused by the presence of random patterns in larger sequence sets. When both the input sequence dataset and queried motif length increase in size, the resulting random noise dilutes potential signals in the data^28^.

One tactic to surmount this limitation is to reframe motif elucidation as a combinatorial optimization problem, where all conceivable sequence combinations exist in a geometric space of disparately optimal motifs. Combinatorial techniques have been shown to more accurately detect subtle signals in noisy sequence datasets, though their use in discovering motifs has been quite limited thus far^29, 30^. With this in mind, we designed a combinatorial heuristic for uncovering hidden DOMs within the haystack of next-gen immune repertoire sequencing data.

Our “Haystack Heuristic” is a DOM discovery technique that is less sensitive to excessive sequence noise. The heuristic implements local search optimization^31^ to locate DOMs within large antibody datasets. Upon design, we tested the heuristic on an assembly of over nine million blood-extracted antibody sequences. The sequences contained the partial B-cell repertoire of 97 distinct individuals, 51 of whom are MS patients. We processed this data with the aforementioned Haystack Heuristic and discovered a DOM, which appears in two-thirds of all MS patients and in none of the healthy controls.

## Materials and Methods

### Generating the Input Sequence Dataset

We performed high-throughput sequencing of immunoglobulin heavy-chain variable-region (IgG-VH) transcripts on peripheral blood samples obtained from 51 multiple sclerosis patients and 46 healthy individuals. The samples were processed and sequenced using our previously published protocol^25^.

#### Sample Collection

We obtained peripheral blood from a total of 51 MS patients and 46 healthy control (HC) volunteers following informed consent. A diagnosis of MS was based on the latest diagnostic criteria for MS^32^. 46 out of 51 patients had relapsing remitting MS. 14 of the patients were treated with Copaxone; 11 were treated with Interferon. Four were treated with Tsyabiri; One was treated with Rituximab; 21 patients remained untreated. Peripheral blood was obtained via standard venipuncture. Peripheral blood mononuclear cells (PBMC) were isolated using a Ficoll gradient, red blood cells lysed and PBMC washed in phosphate buffered saline (PBS) containing 1% BSA. These studies were approved by the institutional review board of the University of California, San Francisco and informed consent was obtained from patients before CSF/PB collection. All methods were performed in accordance with the relevant guidelines and regulations.

#### Unbiased Ig mRNA amplification and Ig repertoire sequencing

In brief, Total RNA was isolated from PBMC (RNeasy mini kit, Qiagen) and RNA quality was assessed using an Agilent Bioanalyzer. Total isolated RNA was reverse transcribed (SMARTerTM RACE, Clontech), and approximately 27% of each cDNA reaction was used for IgG-VH amplification via PCR using SMARTerTM RACE 10X Universal primer mix (Clontech) and an IgG specific 3′ primer (5′-GGG AAG ACS GAT GGG CCC TTG GTG G-3′) for 31 cycles following the manufacturer’s recommendation. In addition to the IgG-specific portion, reverse primers also contained the Lib-L specific adaptor (454 sequencing, Roche) and barcode sequences. Barcoded IgM-VH (~715 bp) and IgG-VH (~640 bp) transcript libraries were purified using AMPure XP (Beckman Coulter Inc.), quantified using PicoGreen (Life Technologies) and normalized to 1 × 10^9 ^molecules/μl. Uniquely barcoded samples were combined in pools of normalized IgG-VH amplified libraries and subjected to emulsion PCR and unidirectional sequencing using the GS FLX Titanium Lib-L chemistry (454 Sequencing, Roche).

#### Analysis

The raw sequence output was analyzed using the VDJFasta algorithm^20^; each sequence was annotated with IGHV and IGHJ germline segments and the amino acid sequence of the Complementary Determining Regions (CDRs). The CDR3 amino acid sequence was used to pick a proper reading frame for nucleotide translation. All translated sequences with a v-gene, j-gene, and CDR3 assignment were stored in a MySQL database. Within that database, the total sequence count was equal to 9,212,022 (Supplementary Table S1 categorizes sequence counts by patient, Supplementary Table S7 shows the lane and run information for each individual). Among these 9 million sequences, 1,531,415 unique CDR3s were present. The MySQL dataset was used as direct input into the Haystack Heuristic.

### The Haystack Heuristic

**Figure 1:**
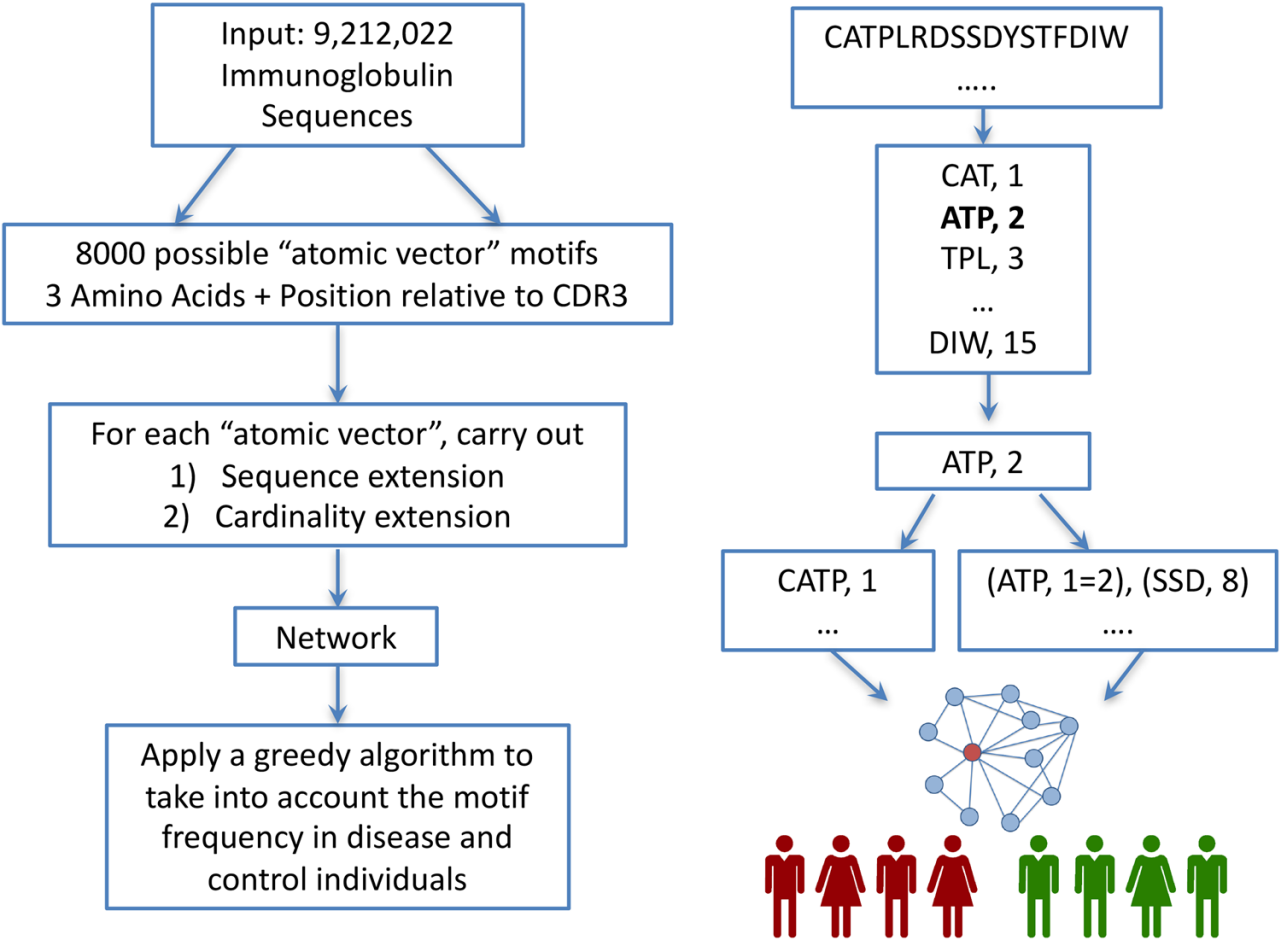
Methodology Overview and Example. (**A**) We start with a set of over 9 million immunoglobulin sequences in amino acid space. The sequences are then broken down to 8,000 (20^3) atomic vectors of size three. For each of the possible vectors we carry out up to three sequence and cardinality extension steps and apply a greedy algorithm to examine the presence of the motif in the MS and healthy individuals. The traversal is stopped if it is not promising based on a threshold. (**B**) Schematic illustration of constructing atomic vectors from a single read sequence. See Haystack Heuristic Overview in the Methods section for a detailed explanation.

The overview of our approach is shown in Fig. 1. We start with a set of over 9 million immunoglobulin sequences in amino acid space. The sequences are then broken down to 8,000 (20^3) atomic vectors of size three. For each of the possible vectors we carry out up to three sequence and cardinality extension steps and apply a greedy algorithm to examine the presence of the motif in the MS and healthy individuals. The traversal is stopped if it is not promising based on a threshold.

### Haystack Heuristic Overview

The Haystack Heuristic searches for position-specific gapped amino acid motifs, short subsequences that are present at specific sequence coordinates. Explicit Motif Subsequences (EMSs) within each gapped motif are linear sequence elements that are separated out by gaps of unspecified sequence characters. Position-specificity arises from the mapping of every EMS to a location in an antibody sequence. Thus, a gapped motif is a vector composed of the EMS itself and an associated antibody coordinate (Fig.1b), the presence of which can be assessed in an IgG-VH sequence at a specified location with respect to the beginning of the CDR3 region. If all the motif vector elements are found within an antibody sequence, then the sequence is treated as a match to the input motif.

For the purpose of our analyses we define an “atomic vector” as the shortest allowable motif. The atomic vector contains a single element, indicating that no gaps are present. That single atomic element is comprised of a sequence coordinate and a short EMS of length ***three***, where ***three*** is the standard minimum length of a short linear motif. Thus, an atomic vector is an amino acid triplet that matches a unique antibody sequence location. The triplet may be extended by adding a single new character to either of its endpoints, resulting in quadruplet motif. Iteratively applying this “EMS extension” will eventually yield a new vector motif of length ***k*** and cardinality ***one***. In this manner, we are able to transform any inputted atomic vector into a gapless motif of length ***k***. Inserting a gap into that motif requires us to increase its vector dimension from ***one*** to ***two***. The simplest way to carry out this “cardinality extension” is to append a new atomic vector to the existing vector motif. Thus, cardinally extending a gapless motif of length ***k*** will produce a single-gapped motif that contains two explicit subsequences of length ***three*** and ***k***. Iteratively applying cardinality extension will eventually yield a motif containing ***n*** gaps and ***n*** **+** ***1*** dimensions. Afterwards, the length of all its ***n*** **+** ***1*** explicit subsequences may be expanded using EMS extension.

EMS extension and cardinality extension (Fig. 1b) allow us to construct complex motifs from simpler input. Starting with a plain atomic vector, we may apply a series of extensions to develop new motifs of higher complexity. Each step within that series represents a physical transition between a lower-order and higher-order motif. For example, suppose we initialize our motif as a singe atomic vector “CAT”, located at coordinate position one. The sequence and position of the vector can be encoded symbolically as (CAT, 1). Using a single EMS extension, a proline may be added to the right-most position of the vector, resulting in new motif of (CATP, 1). Afterwards, a cardinality extension is used to add an additional motif component, SSD at position eight, thus resulting in an updated motif of (CATP, 1), (SSD, 8). Next, an EMS extension extends an arginine to the left-most position of the triplet subsequence SSD, while a second CAT subsequence is cardinally extended at position 15 of the motif. The result is a newly created motif of (CATP, 1), (RSSD, 7), (CAT, 15). Thusly, only four extension operations are required to adequately construct a two-gapped 11 amino acid long motif. That entire process of construction may be represented as a series of physical transitions within a single pathway: (CAT, 1) = > (CATP, 1) = > (CATP, 1), (SSD, 8) = > (CATP, 1), (RSSD, 7) = > (CATP, 1), (RSSD, 7), (CAT, 15). These transitions directly correspond to a shift between neighboring vertices in a unidirectional network. In essence, all motifs exist as nodes within a network whose edges signify EMS and cardinality extensions. Therefore, progressively extending new motifs from simpler input is directly analogous to a directed traversal across a network of motifs.

Furthermore, we propose a network-based motif representation, which permits us to run a network search for DOM motifs. The search initializes at a single node exemplifying an atomic vector. Then, in a breadth-first-search (BFS) approach, the neighbors of the root node are inspected and evaluated. These neighboring motifs are tested against the central dataset of antibody sequences. The sequences matching to each neighbor are aggregated by the individual people from whom they were obtained. Next, all neighboring motifs are marked by the number of patients and healthy controls to which they are a match. If a given neighbor matches the majority of disease-afflicted patients, while also failing to match any of the healthy controls, then we have discovered a DOM and the search is over. Otherwise, the neighbors are assessed on their ability to differentiate disease-afflicted patients from healthy controls. Nodes whose differentiation score falls above a specified threshold undergo a BFS analysis of their own. New neighbors are iteratively obtained and the search space is gradually extended until a DOM is finally found or the search reaches a stipulated endpoint.

Network search capacity to uncover DOMs in data is dependent on the quality of our starting three-character motifs. Triplets situated closer to a DOM in network space serve as better starting nodes then other more isolated vertices. We may therefore enhance our DOM-detecting capabilities by traversing the network from several starting locations. Parallelized search execution on multiple inputs raises the overall yield of useful results. However, such useful results are contingent upon the proper selection of suitable starting motifs. Under ideal conditions, the set of initial motifs would match to every plausible atomic vector. Unfortunately, such breadth of coverage is not computationally viable. Instead, we may settle for an atomic vector subset that contains each conceivable three-character EMS. There exist 8,000 (20^3) three-character amino acid combinations, each of which can be transformed into a full atomic vector by assigning it an optimal coordinate using a preset optimization function. The resulting array of 8,000 vectors may then serve as input for a parallelized network search. The scope of that search should suffice to uncover DOMs hidden in the network.

Our Haystack Heuristic implements the network search procedures discussed above. Many of the outlined steps capitalize on built-in database infrastructures to swiftly match motifs against efficiently stored sequences in memory. Effectively, the Heuristic runs a network search using queries to the database in which the sequence data is deposited. This allows all users to integrate that search within their existing database schema.

The next two subsections discuss all preliminary input settings, as well as algorithmic steps for Heuristic execution.

### Preliminary Input Settings

These specifications must be set prior to running the Heuristic.

#### Categorical Assignment

A “Diagnosis” column in the database categorizes people into individual diagnostic groups. The Haystack Heuristic specifies that all diagnosed individuals must fall into one of two categories; ***A*** or ***B***. Categorical assignment is determined by the diagnosis column. Category ***A*** may be assigned to any diagnoses, including a lack of disease. All diagnoses not in category ***A*** will automatically fall into category ***B***. The total count of people within each respective category is defined as ***|A|*** and ***|B|***.

#### DOM-determining Threshold Selection

Given a motif ***M***, we query the motif against the antibody sequences, grouping the results by the unique individuals from whom the matching sequences were obtained. Afterwards, we count the number of motif-matching individuals from each of the two categories ***A*** and ***B***. These counts are represented by variables ***a*** and ***b***, respectively. If ***b*** is equal to zero, then ***M*** may be considered a potential candidate for DOM categorization. Whether or not ***M*** is actually a DOM is determined by the DOM-determining threshold ***Dt***. Motif ***M*** is considered a DOM only if ***a*** **/** ***|A|*** ***≥*** ***Dt*** and ***b*** **=** ***0***. Thus, threshold ***Dt*** must be assigned a value between zero and one prior to running the Heuristic. For the purposes of solving the base-case problem, we assume the motif only exists in the MS population. The possibility of a low-frequency motif match in the controls is discussed in final paragraph of Assessing the Statistical Significance of Discovered Dom Motifs, and is analyzed rigorously in Supplementary Materials S3.

#### Separation Threshold Selection

For any ***M***, we define a separation score ***S***_***M***_ where ***S***_***M***_ = ***a*** − ***b***. The ***S***_***M***_ parameter measures motif preference for category ***A*** over category ***B***. We set a separation threshold, ***St***, to evaluate a given ***S***_***M***_. If ***S***_***M***_ **>** **=** ***St***, then motif ***M*** remains a candidate for network search. Otherwise, neither that motif nor any of its neighbors will undergo additional analysis. ***St*** values are further discussed in the Testing the Heuristic of the Database section of Methods.

#### Gap Count Specification

We define ***Gc*** as the maximum number of gaps that can occur within our outputted motif. As a result, no starting motif may undergo more than ***Gc*** cardinality extensions. ***Gc*** values are further discussed in the Testing the Heuristic of the Database section of Methods.

#### Running the Haystack Heuristic

Once the preliminary input settings have been specified, the Heuristic is ready to be implemented using the following series of steps:

#### Triplet Extraction

The Heuristic scans all full antibody sequences in order to tally the total occurrence of every unique three-character triplet within the sequence database. Triplets occurring less than Dt*|A| instances are ignored. The remaining triplets are inputted into Step 2.

#### Atomic Vector Generation

Each triplet is transformed into a new atomic vector by selecting a coordinate that maximizes match variable ***a*** (for a detailed discussion of optimal coordinate selection, see Supplementary Materials S1). All vector position coordinates are defined relative to the endpoints of the CDR3 within the triplet-matching antibody sequences. Vectors whose separation score falls below the specified ***St*** do not undergo further analysis.

#### Non-gapped EMS Extension

The database is searched for all non-gapped four-character motifs whose sequences and positions overlap with existing atomic vectors. After ***St*** filtration, all remaining four-character motifs are appended to the atomic vector list. The result is list ***L***, which contains short, non-gapped motifs.

#### Recursive Restriction

Each ***M*** in list ***L*** is queried against the database. All sequences matching a given ***M*** are transferred to a new, temporary database ***dM***. Afterwards, Steps 1–3 are repeated on contents of ***dM***. The resulting list ***L’*** contains non-gapped motifs that may be merged together with ***M***.

#### Motif Merging

Each motif ***M*** is associated with a non-gapped motif list ***L’***. Every motif ***M’*** in ***L’*** contains a single motif vector element. That element may overlap with an existing element of ***M***. If so, then the overlapping elements are merged to lengthen a subsequence of ***M***. This is directly analogous to EMS extension. If there is no overlap, then the element of ***M’*** may be appended to the other elements of ***M***, thereby resulting in cardinality extension. If the merged motifs do not overlap, then this action is the same as the cardinality extension. If they do overlap, then this is analogous to the EMS extension.

#### Iteration

***L*** is replaced by a list of all newly extended motifs. The Heuristic terminates if either a DOM is discovered or the maximum gap length in ***L*** corresponds to ***Gs***. Otherwise, ***L*** is resubmitted to Step 4 of the Heuristic

### Assessing the Statistical Significance of Discovered DOM Motifs

Suppose we encounter DOM **D** with ***a*** matches to ***|A|*** category ***A*** patients and zero matches to ***|B|*** category ***B*** patients. We might assume that if ***P***_***A***_(***D***) is the probability of matching ***D*** to a category ***A*** patient, and if ***P***_***B***_(***D***) is the probability of matching ***D*** to a category ***B*** patient, then ***P***_***A***_(***D***) must be significantly greater than ***P***_***B***_(***D***). However, this might not necessarily be the case. We must consider the possibility that ***D*** is a homogenous motif for which ***P***_***A***_(***D***) is equal to ***P***_***B***_(***D***). If ***D*** is homogenous, then it will match all patients will equal likelihood, regardless of category. Under such circumstances, the presence of ***D*** in the Heuristic results will be a purely random anomaly. Thus, in order to demonstrate statistical significance, we must show that the likelihood of encountering a homogenous DOM is significantly low for our results.

In order to do so, let us first assume that ***D*** is a homogeneous motif that matches all patients with an equal probability of ***p***_***x***_. The probability that ***D*** is a DOM with ***a*** total matches may be represented by function ***P***(***p***_***x***_, ***a***, ***|A|***, ***|B|***). We are unable to compute ***P***(***p***_***x***_, ***a***, ***|A|***, ***|B|***) directly, because the value of ***p***_***x***_ is unknown. However, we are able to calculate the maximum possible value of ***P***(***p***_***x***_, ***a***, ***|A|***, ***|B|***), thereby representing maximum possible likelihood of encountering a homogenous DOM. Our calculations in Supplementary Materials S2 demonstrate that ***P***(***p***_***x***_, ***a***, ***|A|***, ***|B|***) ≤ ***ML***(***a***, ***|A|***, ***|B|***), where ***ML***(***a***, ***|A|***, ***|B|***) **=** $$(\begin{array}{c}|{\boldsymbol{A}}|\\ {\boldsymbol{a}}\end{array})$$(***1*** − ***w***)^***a***^(^1^^/^^w^^−^^1^)***w*** ^***a***^ and the variable ***w*** is equal to ***a*** **/**(***|A|*** **+** ***|B|***).

Using the formula ***ML***(***a***, ***|A|***, ***|B|***), we are able to show that the likelihood of randomly encountering certain homogenous DOMs is exceedingly low. However, that in of itself is not enough to demonstrate significance. The Haystack Heuristic traverses across millions of antibody motifs, any of which may be a homogeneous DOM. Thus, we must show that ***E***(***C***, ***a***, ***|A|***, ***|B|***) < ***1***, where ***E***(***C***, ***a***, ***|A|***, ***|B|***) the expected count of random homogenous DOMs that match ***a*** or more patients across a set of ***C*** total motifs. Using our previous calculations it is trivial to show that ***E***(***C***, ***a***, ***|A|***, ***|B|***) ≤ C*$\sum _{{\boldsymbol{i}}={\boldsymbol{a}}}^{|{\boldsymbol{A}}|}{\boldsymbol{ML}}({\boldsymbol{i}},|{\boldsymbol{A}}|,|{\boldsymbol{B}}|)$. Thus, if the Heuristic traverses ***C*** motifs and elucidates a DOM defines by ***a***, then we must calculate the Maximum Expected Value ***Mev*** where ***Mev*** = C*$\sum _{{\boldsymbol{i}}={\boldsymbol{a}}}^{|{\boldsymbol{A}}|}{\boldsymbol{ML}}({\boldsymbol{i}},|{\boldsymbol{A}}|,|{\boldsymbol{B}}|).$ If the ***Mev*** is significantly less than one, then we may deem the DOM statistically significant. Otherwise, must we must discard the DOM as unreliable result.

A low ***Mev*** indicates that ***P***_***A***_(***D***) is unlikely to equal ***P***_***B***_(***D***). It does not however, indicates that ***P***_***B***_(***D***) necessary equals zero. As discussed in Supplementary Materials S3.1, the presence of a statistically significant DOM simply indicates ***P***_***A***_(***D***) > ***P***_***B***_(***D***). Thus, the DOM might still appear within certain category ***B*** patients, though with a lesser frequency relative to all category ***A*** matches. Though ***P***_***A***_(***D***) and ***P***_***B***_(***D***) may not be computed directly, it is in our interest to show that ***P***_***A***_(***D***) is significantly greater than ***P***_***B***_(***D***). We have thusly developed a protocol for estimating the maximum value of ***P***_***B***_(***D***)**/** ***P***_***A***_(***D***). That protocol is presented in Supplementary Materials S3.2. As presented in Supplementary Materials S1, optimizing on a category-blind selection function (total # of people matched – 51)^2 still leads to the prime motif getting discovered.

### Testing the Heuristic on the Database

We ran the Heuristic on the sequence data stored within the MySQL database (statistics are shown in Supplementary Table S1) two times. In the preliminary run, MS patients were assigned to category ***A*** while Healthy Controls were assigned to category ***B***. In the secondary run, these categorical assignments were reversed. The ***Mev*** was calculated for all discovered DOMs in order to evaluate significance. The input parameters for the both Heuristic runs were set as follows; ***Dt*** **=** ***0***.***6***, ***St*** **=** ***15***, ***Gc*** **=** ***2***.

### Code and Data Availability

An optimized example implemented in python in order to make the methodology more reproducible is available at https://github.com/lapeltsin/HaystackHeuristic. The data is available at: http://www.immport.org/immport-open/public/study/study/displayStudyDetail/SDY1043.

## Results

### Searching for MS Specific Motifs using the Haystack Heuristic

**Figure 2:**
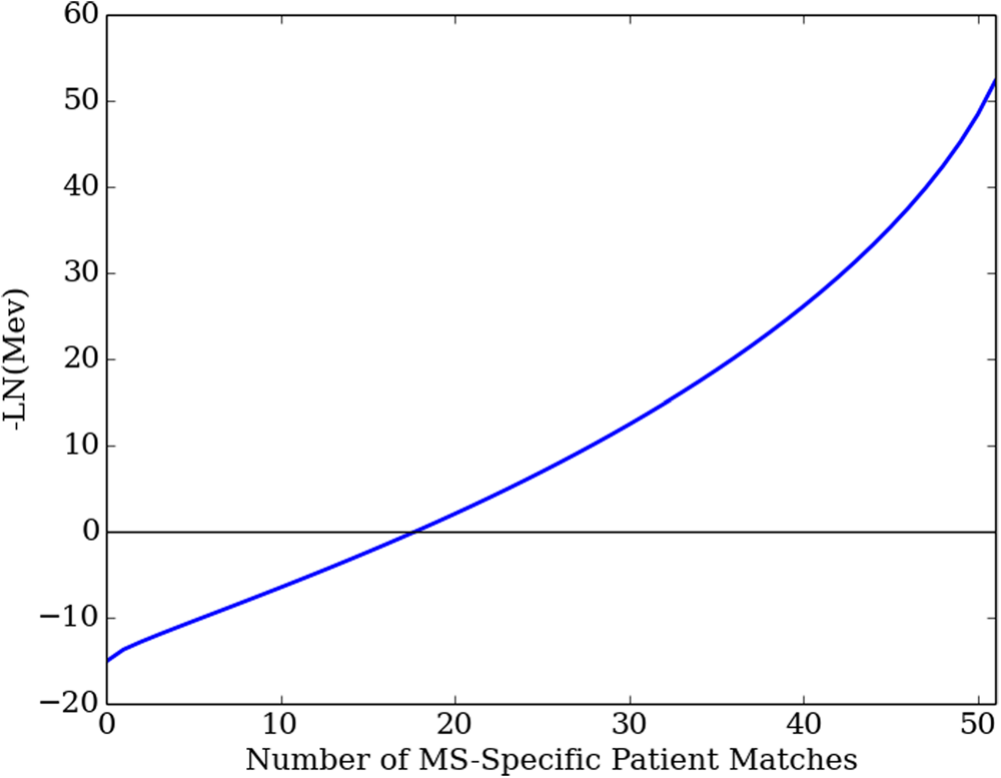
The -LN(Mev) is plotted for all possible DOM matches counts, ranging between zero and 51 MS patient matches. The plot is negative for match counts below a total of 20 patients, indicating that a DOM match to 20 patients or less may potentially occur at random. The plot is significantly greater than zero at 30 patient matches, which is our cutoff threshold for DOM categorization.

The Heuristic traversed 2,743,571 motifs and identified 171 MS-specific DOMs, which are listed in Supplementary Table S2. The DOM patient match counts ranged from 30 to 35 MS patients (Table 1). The DOM ***Mev*** quantities ranged from 3.98E-06 to 7.49E-09, (Fig. 2), indicating that the DOM results were not occurring by random.

**Table 1:** Each row of the table corresponds to DOM motifs associated with a patient match count ranging 30 and 35.

Patient Match Count	^#^Unique Motifs	Motif with Maximum Hit Count	Maximum Hit Count
35	6	(TNE, 14), (DTA, 6), (CAR,0)	104
34	55	(TNE, 14), (VYYCAR, 3)	93
33	1	(SKN, 21), (YLT, 16), (PES, 10)	62
32	6	(YLTN, 16), (DTA, 6), (CAR, 0)	87
31	37	(YLTN, 16), (VYYCAR, 3)	79
30	66	(RDN, 24), (YLTN, 16), (CAR, 0)	74

The patient match counts are listed in the first column. The second column contains the number of DOM motifs associated with each match count. The third column contains the DOM motif that matches the maximum number of sequences for a particular match count. The number of sequences matched by the third-column column motif is listed in the final column of the table. All motifs are stated in a parenthesis notation where (*S*, *x*) represents an non-gapped subsequence *S* that is located *x* amino acids to the left of the CDR3. The Prime Motif appears in row 1, column 3 of the table.

The 171 MS-specific motifs appeared to be similar in structure. All 171 motifs contained a threonine located 14 amino acids to the left of the CDR3. In addition, 66% of the motifs contained a cysteine followed by an alanine at the start of the CDR3. These similarities implied that all MS-specific motifs were matching to an overlapping set of sequences. In order to investigate motif-match sequence overlap, we examined the motif with the maximum number of sequence and patient matches. This “Prime Motif” matched to 35 patients and 104 antibody sequences. The Prime Motif matched sequences were analyzed for overlap between the matching sequences for the remaining 170 motifs. All 170 motifs matched to sequences that overlapped with 104 the Prime Motif antibody matches. The fraction of overlapping sequences ranged from 36% to 100% across all discovered motifs (Supplementary Table S2). On average, 75% of motif matches overlapped with the 104 Prime Motif-targeted sequences. Therefore, we concluded that the Prime Motif held properties that corresponded to the structure of all other motifs. As a result, the Prime Motif underwent additional analysis.

### Exploring the Prime Motif

**Figure 3:**
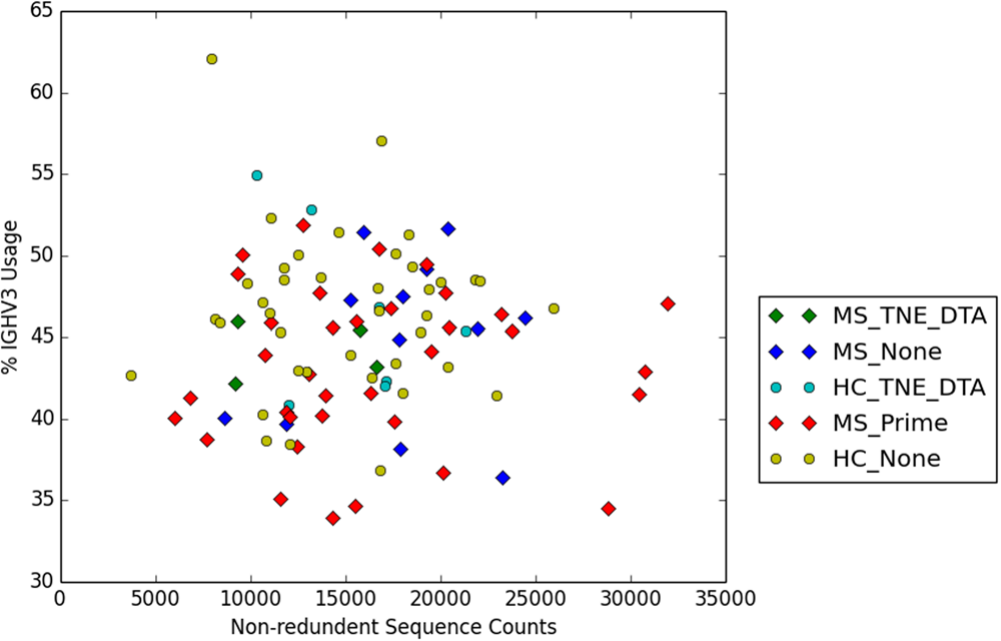
All 51 patients and 46 Healthy Controls are represented in this 2D plot. The x-axis contains the nun-redundant patient sequence counts, obtained from column three of Supplementary Table 1. The y-axis contains the IGHV3 usage percentages obtained from column two of Supplementary Table 5. The data point shapes are determined by patient type. MS patients are represented by diamonds. Healthy Controls are represented by circles. The data points are further subcategorized by match type. Prime motif matches are categorized as “Prime.” TNE and DTA matches are categorized as “TNE_DTA.” All other matches are categorized as “None.” Though a few MS outliers are present in the plot, most patient data points appear to overlap regardless of categorization.

We characterized the Prime Motif as “(TNE,14),(DTA,6),(CAR,0)”, using the parenthesis notation introduced in Table 1. This motif was composed of three separate subsequences; TNE, DTA, and CAR. The subsequence starting coordinates were located respectively at 14, three, and zero positions to the left of the CDR3. Each of the 35 motif-associated repertoires held between one and ten Prime Motif matching sequences, the variable regions of which were all IGHV3 (Supplementary Table S3). The antibody sequence matches to the Prime Motif were not dependent on the total patient sequence counts or IGHV3 variable region usage (Fig. 3). Therefore, we had reason to believe that the presence of the Prime Motif was not merely an artifact and that its role in Multiple Sclerosis might somehow be physiologically significant.

We further probed the nature of the Prime Motif by investigating its individual subsequence components. Among the three subsequences, only CAR was found within the antigen-targeting loop CDR3. CAR, however, represented an unmodified germline segment known to commonly occur at the starting point of the CDR3 region^33^. Therefore, the CAR component did little to account for the uniqueness of the Prime Motif. Motif specificity appeared to be a product of subsequences TNE and DTA. Querying the database against matches to just these two components yielded a total of 204 antibody sequences. The 204 sequences were distributed across 46 individual repertoires (Supplementary Table S4). Four MS patients and seven Healthy Controls matched both to TNE and DTA, but not to the entire Prime Motif. Thus, 80% of the repertoires containing TNE and DTA mapped to an MS diagnosis, implying that the presence of the two components factored into MS selectivity. Also, as with the Prime Motif, the two selective components were not influenced by repertoire size or IGHV3 usage (Fig. 3).

**Figure 4:**
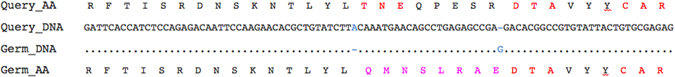
The igBlast alignment of a Prime Motif matching subsequence with its germline origin. The subsequence includes all three motif components; TNE, DTA, and CAR. These components have been highlighted in red. The nucleotide contents of the queried input sequence are listed on the line marked Query_DNA. The proper amino acid translation of the query is included on the line marked Query_AA. The third line, labeled as Germ_DNA, features an alignment between the query nucleotides and the germline sequence IGHV3-53. Each dot in that alignment represents a nucleotide that has not mutated from the germline origin. The line as marked as Germ_AA contains the amino acid translation of the germline nucleotides. Based on the alignment, the query is identical to the germline, except for a single insertion and a single deletion. These indels, which are highlighted in blue, serve as endpoints of components TNE and DTA. The indels result in a localized reading frame shift, which transforms the germline subsequence QMNSLRAEDTA (highlighted in purple) into a Prime Motif associated subsequence TNEQPESRDTA.

**Figure 5:**
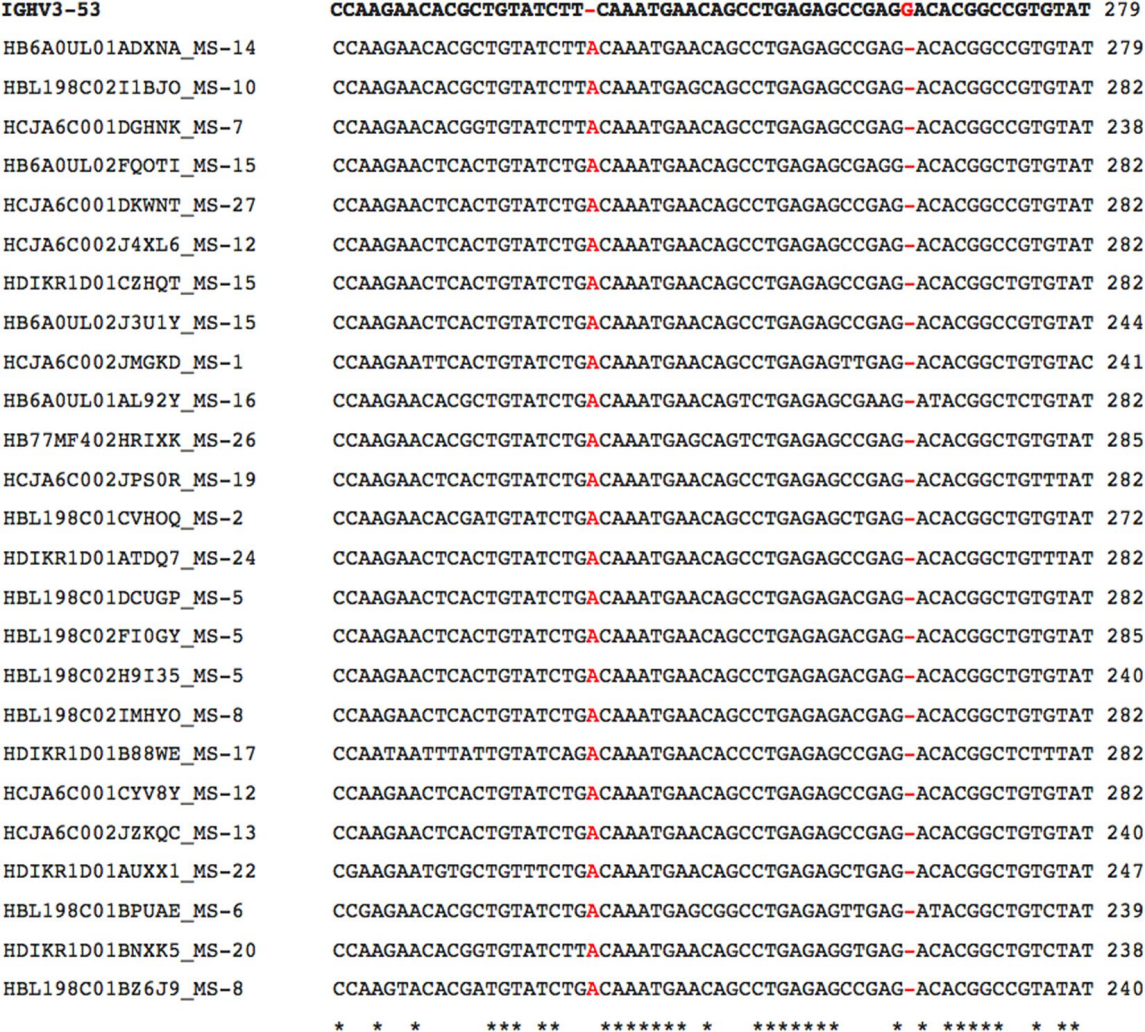
A subset of Prime Motif matching nucleotides have been aligned to the IGHV3-53 germline sequence. The visualized segment of the resulting multiple alignment corresponds with the FR3 region of the antibody sequence. All other lines contain subsequences obtained from Prime Motif matching antibodies. Insertions and deletions in the multiple alignment are highlighted in red. A single adenine insertion is present in all sequences within the input nucleotide subset. The insertion is followed by a single deletion that occurs within the space of 24 nucleotides.

We scrutinized more closely how the TNE and DTA components developed from nucleotide mutations. The nucleotide sequences of all 104 Prime Motif associated antibodies underwent analysis using igBlast, a computational tool for identifying the relationships between antibodies and their germline origins^34^. The inspection of a sample igBlast alignment revealed an adenine insertion in the nucleotides of the TNE component (Fig. 4). In addition, a deletion flanked the first position of the DTA component, which itself remained identical to its germline counterpart. Thus, it appeared that the two selective components marked the endpoints of a 24 base long frameshift mutation. The multiple alignment of a motif-matched sequence subset helped confirm that a localized reading frame shift was present in the framework three (FR3) region of the antibodies, relative to the IGHV3 gene (Fig. 5). FR3 insertions and deletions have previously been observed in B-cell sequences^35, 36^. These insertions and deletions are believed to be compatible with antigen-targeting antibody functions^36^.

Based on our alignment observations, we supplemented the initial Prime Motif with an additional indel requirement. The indel requirement stipulated that a TNE-adjoined insertion and a DTA-adjoined deletion must be present in all Prime Motif matching sequences. We tested this constraint on the 104 sequences obtained from the initial Prime Motif. 101 of the original 104 sequences complied with our indel requirement. These indel-associated sequences remained distributed across 35 unique patient repertoires (Supplementary Table S6).

### Searching for HC Specific Motifs using the Haystack Heuristic

The Heuristic traversed 175,426 motifs aiming to find Healthy Control specific motifs. No DOM sequences were found in that traversal. Additionally, the Heuristic did not encounter any motifs that matched to zero MS patients. Instead, a total of 19,104 motifs matched to a single MS patient. Each of these single-MS motifs matched between 15 and 20 Healthy Controls. All match counts for the single-MS motifs fell below the DOM-determining threshold of 60%. Thus, further traversal of the single-MS motifs would not lead to eventual DOM discovery. We concluded that control-specific motifs were not present in our repertoire sequence dataset.

### Comparison to Other Approaches

In our efforts to investigate alternate approaches to biomarker motif discovery, we used the HOMER (Hypergeometric Optimization of Motif EnRichment) Software Suite^37^ to search for nucleotide motifs that were over-represented in the patient sequence set relative to the healthy controls. The HOMER output contained 25 over-represented motifs, ranging from eight to twelve nucleotides in length. Plotting the percentage of MS sequences matched by each motif relative to the percentage of matched Healthy Controls revealed a strongly linear relationship (Fig. S2). The regression slope of 0.68 indicated that the HOMER motifs were not significantly over-represented in the MS data, thus limiting their potential diagnostic utility.

As a substitute approach to elucidating out a predictive signal from our sequence repertoires, we attempt to train a model to predict the presence of MS from sequence data. For model building purposes, we used the random forest learning algorithm, was shown to have the capability of handling a large number of input variables while avoiding model overfitting^38^. To generate the model, a feature set was constructed for each of the patients and healthy controls. Each set of features for each sample corresponded to the frequencies of amino acid occurrences relative to the residue positions used in the Haystack Heuristic motif vectors. The Scikit-Learn machine learning module^39^ was then used train a model to differentiate between patients and Healthy Controls based on the positional residue frequencies. Five-fold cross-validation was applied to evaluate the model’s predictive potential. The F-measure, a commonly used evaluation metric computed from the geometrical mean of precision and recall, was equal to 0.63, indicating that the random forest model itself had very little predictive power.

## Discussion

The Haystack Heuristic proved successful at its fundamental task of uncovering disease-related patterns in a vast noise-prone sequence dataset. After processing millions of Ig-VH sequences, the Heuristic was able to detect an MS-associated motif matching to less than 0.01% of analyzed sequences. Based on the calculated ***Mev***, this motif is unlikely to have occurred by random. Furthermore, a search for HC-specific motifs did not yield any viable results; thereby implying that the Heuristic is sensitive to input data and is not prone to random output. Our findings suggest that a genuine separating signal is present among MS B-cell repertoires.

Two separate runs of our Haystack Heuristic were performed. The first run, optimized for MS patient matches, traversed approximately 2.7 million nodes in motif network space. The second run, searching for motif enrichment in HC data, traversed approximately 175 thousand nodes within the network. Thus, there was a 15-fold variation in overall network coverage between the two Heuristic executions. However, both runs commenced from an equal of number of initial vertex positions, hence the starting conditions are unlikely to have influenced the final observed divergence. Rather, the results imply that differences in coverage may be caused by the difference in separation scores used to jettison nodes unsuitable for additional traversal. An elevated set of separation scores would account for the extended network scope of the primary Heuristic run. Since the separation scores represent the balance between the number of MS and Healthy Control matches, we can conclude that certain lopsided motifs are present in our processed B-cell repertoires. Such lopsided motifs are found in MS patients with an implicitly higher frequency relative to the Healthy Control data. Inversely, an HC-inclined imbalance does not appear to substantially occur, based on the limited network coverage of our second Heuristic execution. Consequently, we conclude that the observed motif disparity is an illness-related phenomenon. However, additional work such as B-cell repertoire sequencing of NMO patients will be necessary to determine whether the motif is an MS-specific feature able to differentiate one CNS autoimmune disease from another.

The Haystack Heuristic identified a set of motifs whose presence is more frequent within the blood of MS patients. Among that set, the Prime Motif particularly stands out. The total observed frequencies of Prime Motif occurrences within the MS patients and the Healthy Controls are respectively, 68% and 0%. However, the observed frequency of 0% does not guarantee that the Prime Motif is always absent from within all healthy repertoires. It is quite possible that the Prime Motif is occasionally present in Healthy Controls, but with a lower frequency relative to all MS-linked occurrences. Confirming the precise occurrence frequencies may be the goal of future studies on replication cohorts and with increased sequencing depth. Nevertheless, our calculations provide strong evidence that the Prime Motif itself is substantially more likely to occur within the repertoire of disease-afflicted patient (Supplementary Materials S3.3). Thus, the Prime Motif might serve as a useful biomarker for more nuanced or possibly earlier MS diagnosis. At the very least, the role of the Prime Motif in at risk populations and in context of autoimmune disease progression should be examined in more detail. Some extensions of this work would include applying machine learning classifier approaches for disease diagnosis, however further utility of such methods needs to be evaluated.

Our present understanding of the Prime Motif is limited to sequence-based interpretations and its biological relevance remains subject to future studies. However, it is conceivable that the Prime Motif resulting from a productive frame shift in the Ig-VH FR3 region may directly alter structural and consequently binding properties of BCR. Furthermore, the FR3 region has been proposed to be directly involved in immunoglobulin interaction with antigen targets, for example between Staphylococcal Protein A and FR3 region of IGHV3-enconded receptors^36, 40^. Thus, in an autoimmune disease with prominent B cell involvement, the Prime Motif may not only serve as biomarker but may also aid in the understanding of immune mechanisms involved in the etiology of demyelinating CNS pathology. In this regard, our findings raise additional questions. In our study, the overall count of motif-matched sequences is low, suggesting that none of the associated peripheral blood B cells were actively undergoing expansion during patient sample collection. While we cannot exclude the possibility that a Motif-triggered inflammatory response occurred at an earlier stage of disease onset, future studies with increased sequencing depth and at different stages of MS are necessary to address this question. Furthermore, alternate samples from non-MS autoimmune patients would help us examine the role of the Prime Motif described here or similar ones in more general autoimmune responses. We will continue to explore the validity of the Prime Motif as biomarker and pathway to biological explanations of autoimmunity.

While our Heuristic was successful at identifying a possible differentiating motif, this first-pass implementation may also lack in several aspects. For example, the search algorithm is dependent on a greedy, hill-climbing technique that may, or may not discover the most optimal differentiating motif. The addition of a stochastic search component would be a nice future extension of the approach presented here resulting in increased search-space coverage. Furthermore, our motif-vector model currently relies upon exact amino acid matches. Future iterations of the heuristic will need to take into account partial matches based on amino acid similarity and polarity. The current scoring function does not evaluate the quantity of motif-matching sequences within each sampled patient. Adding a per-patient sequence match component might help push the heuristic towards discovering more immunologically active disease motifs. Finally there are issues with detection limit that should be considered - at greater depth there will always be some false positives of any motif occurring in the negative control population. Likewise at too low a depth too many false-positive motifs will appear due to insufficient sampling saturation of the motif graph. Nonetheless, the current heuristic implementation serves as an adequate baseline for measuring improvements to the algorithm.

In this work, we present the “Haystack Heuristic”, a DOM discovery technique that uses local search optimization to locate motifs within large datasets of antibody sequences. Upon design, we tested the heuristic on an assembly of over nine million blood-extracted antibody sequences with partial B-cell repertoire of 97 distinct individuals, including 51 MS patients as well as healthy controls. The Haystack Heuristic was applied to discover a DOM, which appears in two-thirds of all MS patients studied here and in none of the healthy controls and might, after further necessary validation in replication cohorts, and in comparison to other systemic or CNS autoimmune diseases, serve a diagnostic biomarker for MS.

## Electronic supplementary material

Supplementary PDF File
