# Supplementary material for: A Haystack Heuristic for Autoimmune Disease Biomarker Discovery Using Next-Gen Immune Repertoire Sequencing Data
Source: Sci Rep. 2017 Jul 13;7:5338. doi: 10.1038/s41598-017-04439-5 (PMC5509648; doi:10.1038/s41598-017-04439-5)
Supplement: Supplementary file 1 — Supplementary PDF File [file 41598_2017_4439_MOESM1_ESM.pdf]

A Haystack Heuristic for Autoimmune Disease Biomarker Discovery Using Next-Gen Immune Repertoire Sequencing Data

Authors:

Leonard Apeltsin[1], Shengzhi Wang[1], H.-Christian von Büdingen[1,*], Marina Sirota[3]

Affiliations:

[1] Department of Neurology, UCSF, San Francisco, 94158, USA; [3] Institute for Computational Health Sciences, UCSF, San Francisco, 94158, USA

*Author for correspondence:

H.-Christian von Büdingen, MD
Department of Neurology
University of California, San Francisco
San Francisco, CA 94158 - USA
Email: Hans-Christian.vonBuedingen@ucsf.edu

Supplementary Materials 1

This section discusses the use and selection of Positional Optimization Functions (POFs). We begin by first defining an atomic vector ***V*** as tuple of values ***(T,P)***, where ***T*** is a three-character triplet and ***P*** is its preset position. Given any input triplet ***T***, we need to be able to transform it into a new atomic vector. This can be done by querying the database for matches to ***T*** across all possible positions. From those results, we allocate motif ***Mx*** to every match occurring at unique position ***x***. For each ***Mx,*** we calculate the position-specific match counts a_x and b_x. These variables must next be processed by some specified POF. The POF will scan all instances of ***x*** in order to determine an optimal position ***oP***, which will then be used to transform triplet ***T*** into an atom vector ***(T,oP)***.

Our requirements for POF specification remain open-ended. We have tested several POFs in the implementation of the Heuristic. Our most basic POF is the A-optimization function, which scans motif matches at all ***x*** positions in order to pinpoint the maximum possible a_x. Effectively, A-optimization locates the triplet position that yields the maximum number of matches to category ***A*** individuals. The Heuristic, as described in the paper, implements A-optimization.

An alternate optimization approach is combinatorial optimization (C-optimization), which minimizes $(a_x + b_x - |A|)^2$. In this manner, C-optimization attempts to adjust the overall match counts so that they approximate the original number of category ***A*** individuals. C-optimization is more convoluted then A-optimization; it is explicitly blind to whether selects for category ***A*** or category ***B*** individuals. Our purpose for implementing C-optimization was to test for Heuristic stability relative to the chosen POF.

We ran our Heuristic with A-optimization, as described in the paper. Afterwards, we reran the Heuristic with C-optimization in order to determine how a change of POF would influence DOM output. The DOMs discussed through C-optimization were identical to the DOMs discovered through A-optimization. Both techniques produced the Prime Motif, which we analyze in Section 3.2. From this, we concluded that the final Heuristic output is not exceedingly sensitive to POF specification.

Supplementary Materials 2

Suppose that a given motif ***Mx*** matches any patient with probability p_x. All matches of ***Mx*** to unique patients are independent of each other. Thus, the probability the ***Mx*** matches to m distinct patients is p_x^m. Conversely, the probability that ***Mx*** does not match n distinct patients is $(1-p_x)^n$. Suppose we encounter ***t*** total patients, ***m*** of which match motif ***Mx***, and ***t-m*** of which do not. The probability of this occurring is $P(p_x,m,t-m) = \binom{t}{m}(1-p_x)^{t-m}p_x^m$.

Suppose we encounter Dom ***Dx***, which matches any patient with probability p_x. The match to a of A total patients occurs with probability $P(p_x,a,|A| - a) = \binom{|A|}{a}(1-p_x)^{|A|-a}p_x^a$. Furthermore, because ***Dx*** is a DOM, ***b*** is equal to zero. The probability that all category ***B*** patients will fail to match ***Dx*** is $P(p_x,0,|B|) = (1- p_x)^{|B|}$. It follows that the total probability of encountering DOM ***Dk*** is $P(p_x,a,|A| - a)*P(p_x,0,|B|)$, which equals $\binom{|A|}{a}(1- p_x)^{|A|-a}p_x^a(1- p_x)^{|B|}$ .That formula simplifies down to $\binom{|A|}{a}(1- p_x)^{|A|+|B|-a}p_x^a$.

We represent ***Pr(Mx,a)*** as the probability that a given motif ***Mx*** is a DOM with ***a*** total patients matches. Our calculations show that $Pr(Mx,a) = \binom{|A|}{a}(1- p_x)^{|A|+|B|-a}p_x^a$. All the variables in that formula may be quantifiably obtained, except for p_x. Variable p_x remains an unknown quantity that varies for each motif Mx. Though we are unable to determine an a priori value for p_x, we are able to calculate the p_x quantity that will maximize the value of ***Pr(Mx,a)***. These calculations are discussed in the subsequent paragraph.

In order to maximize ***Pr(Mx,a)***, we must maximize $(1- p_x)^{|A|+|B|-a}p_x^a$ relative to p_x. This is equivalent to maximizing the function $f(p) = (1- p)^x p^y$ relative to p. With some elementary calculus, it is trivial to show that $\frac{df(p)}{dp} = yp^{y-1}(1-p)^x - x(1-p)^{x-1}p^y$. When $\frac{df(p)}{dp} = 0$, equation may be solved for ***p*** to show that $p = y/(x+y)$. Setting ***p*** to $y/(x+y)$ will maximize the value of ***f(p).*** Consequently, setting p_x to equal $a/(|A| + |B|)$ will maximize the value of ***Pr(Mx,a***). Thus, if variable $w = a/(|A| + |B|)$ then $|A|+|B| - a = a(1/w - 1)$, and the maximum value of ***Pr(Mx,a)*** is equal to $\binom{|A|}{a}(1- w)^{a(1/w -1)} w^a$. Therefore, $ML(a,|A|,|B|) = \binom{|A|}{a}(1- w)^{a(1/w -1)} w^a$, where ***ML(a,|A|,|B|)*** represents the maximize possible likelihood that any motif will match ***a*** of ***|A|*** category ***A*** patients and zero of ***|B|*** category ***B*** patients.

Supplementary Materials 3

S3.1 Unequal Frequency Analysis When Category B Matches Hold a Higher Likelihood

Suppose that a given motif ***M*** matches to category ***A*** patients with probability ***p***, and also matches to category B patients with probability p_2. If the ratio of p_2 to ***p*** is ***n***, then $p_2 = np$. Thus, if ***P(p,a,n,|A|,|B|)*** is the probability that ***M*** is a DOM with ***a*** matches given the variables ***p,n,|A|,*** and ***|B|***, then $P(a,n,p,|A|,|B|) = \binom{|A|}{a}(1-np)^{|B|} (1-p)^{|A|-a}p^a$. We have already considered the instance where ***n = 1***. Let us instead consider the case where ***n=g*** and ***g > 1***. Under such circumstances, $gp > p$, $1-gp < 1-p$, $(1-gp)^{|B|} < (1-p)^{|B|}$ and $P(a,g,p,|A|,|B|) \leq P(a,1,p,|A|,|B|)$. As a result, the expected occurrence of a motif where ***n=g*** is less than the expected occurrence of a motif where ***n=1***. Thus, if the ***Mev*** of a DOM is significantly less than one, then we can immediately conclude that ***n*** is not equal to ***g*** and that p_2 is less than ***p***.

S3.2 Unequal Frequency Analysis When Category A Matches Hold a Higher Likelihood

Suppose that that the heuristic searches through C total motifs and discovers w total DOMs. The Mev for each of the w DOMs is significantly below zero. From this, we conclude that the n probability ratio must be less than one within the DOM motifs. Our next goal is to determine the maximum allowable value for ratio **n**. To do so, we assume that C_2 of the visited C motifs contain a probability ratio of $n < 1$. We may dive these C_2 motifs into k different groups where every group j contains X_j motifs with probability ratio n_j. Thus, $\sum_{j=1}^{k} X_j = C_2$, where $C_2 \leq C$. If $V(p,a,n,|A|,B|) = \sum_{t=a}^{|A|} P\ (t,n,p,|A|,|B|)$ then $X_j{*}V(p,a,n_j,|A|,|B|)$ is equal to the expected number of DOM motifs within group j that match at least a patients. Thus, if all our w DOM motifs match to m or more patients, then we can model the equation for expected DOM occurrence as $\sum_{j=1}^{k} X_j{*}V(p,m,n_j,|A|,|B|) = w$.

Let us assume that our k groups are ordered such that $n_{j+1} > n_j$. As a result, n_1 represents the lowest-valued probability ratio among the **k** groups. It is trivial to show that as n_j decreases, the value of $V(p,m,n_j,|A|,|B|)$ must increase. Thus, $V(p,m,n_1,|A|,|B|) \geq V(p,m,n_j,|A|,|B|)$. From this relationship, we may iteratively derive the following sequence of inequalities:

1) $\sum_{j=1}^{k} X_j{*}V(p,m,n_1,|A|,|B|) \geq w$
2) $V(p,m,n_1,|A|,|B|)\ {*}\sum_{j=1}^{k} X_k \geq w$
3) $V(p,m,n_1,|A|,|B|){*}C_2 \geq w$
4) $C_2 \geq w/V(p,m,n_1,|A|,|B|)$
5) $ln(C_2) \geq ln(w) - ln(V(p,m,n_1,|A|,|B|))$
6) $ln(C_2) - ln(C) \geq ln(w) - ln(V(p,m,n_1,|A|,|B|)) - ln(C)$
7) $1 \geq C_2/C$
8) $0 \geq ln(C_2) - ln(C)$
9) $0 \geq ln(w) - ln(V(p,m,n_1,|A|,|B|)) - ln(C)$
10) $ln(V(p,m,n_1,|A|,|B|)) + ln(C) - ln(w) \geq 0$

Let us assume that $F(p,n_1) = ln(V(p,m,n_1,|A|,|B|)) + ln(C) - ln(w)$, were $m,w, C, |A|, |B|$ are all constant values. An alternate function, $M(n_1)$, represents the maximum possible value of $F(p,n_1)$ relative to a constant n_1. This maximum value is directly depended on the maximum value of $V(p,m,n_1,|A|,|B|)$, which we represent as function $Vm(n_1)$. Function $Vm(n_1)$ may be solved numerically by applying a Golden Section Search to $V(p,m,n_1,|A|,|B|)$. Therefore, $M(n_1) = ln(Vm(n_1)) + ln(C) - ln(w)$. Based our tenth inequality, we know that for any valid n_1, function $M(n_1)$ must be greater than or equal to zero. We can also easily show that $M(n_1)$ decreases as n_1 increases in value. Thus, if r is a root for which $M(r) = 0$, then any for any $x > r$, $M(x) < 0$. Effectively, r represents the maximum possible value of a valid n_1. By solving for **r**, we are able to state that $p_2/p \leq r$.

In this manner, we are able estimate the upper bound of the probability ratio for any discovered DOM motif. Given a DOM with parameters ***(m,w, C, |A|,|B|)***, we solve for the root of $M(n_1)$ using common numerical methods. The resulting solution for $M(n_1) = 0$ will give us the maximum possible ratio of DOM match probabilities between category ***B*** and category ***A*** patients.

S3.3 Running an Unequal Frequency Analysis on the MS Sequence Dataset

When implementing the Haystack Heuristic, we assigned ***51*** MS patients to Category ***A***, and ***46*** Healthy Controls to Category ***B***. Afterwards, the Heuristic traversed ***2,743,571*** total motifs and outputted ***171*** DOMs. Thus, $M(n_1) = \ln(Vm(n_1)) + \ln(2{,}743{,}571) - \ln(171)$ for the results in our analysis. We plotted $M(n_1)$ for all values of n_1 ranging from ***.01*** to ***1*** (Supplementary Figure 1). As that plot demonstrates, $M(n_1) = 0$ when n_1 is approximately ***.31***. Therefore, the DOM occurrence frequency within Healthy Controls is at most 31 percent of the DOM occurrence frequency within the MS patients.

Supplementary Materials 4

The Prime Motif was present in 104 sequences, which were distributed across 35 patients. As discussed in Section 3.2, the main differentiating factor in the Prime Motif involved the two selective components TNE and DTA. The selective components matched to 204 sequences, which were distributed across 46 individuals. All antibody matches had originated from the IGHV3 germline. The domination of the IGHV3 germline suggested that the Prime Motif might simply be an artifact of IGHV3 expansion among certain motif-matched individuals. In order to disregard this possibility, it was necessarily to analyze IGHV3 expansion across all repertoires. To do so, we first computed the number of non-redundant sequences across each of our 97 repertoires. Non-redundancy was realized by counting each unique CDR3 exactly once, for every processed repertoire. Afterwards, for every repertoire count, we computed the percentage of non-redundant sequences that were assigned to a specific IGHV3 gene. This percentage formed the IGHV3 usage, which we used to rank the repertoires by their levels of IGHV3 expansion. The ordered IGHV3 usage results are displayed in Supplementary Table 5.

The order of the repertoires in Supplementary Table 5 were clearly not dependent on the presence of the Prime Motif. The first five repertoires within the table all originated from Healthy Controls. Two of these five Healthy Controls contained matches to TNE and DTA. Starting with the sixth position, we began see the Prime Motif matching repertoires, which were evenly distributed across the table. Eight of the final ten repertoires in the table matched to components TNE and DTE. Seven of these repertoires also contained matches to the Prime Motif. Thus, neither the Prime Motif nor the selective components were influenced by IGHV3 usage.

Supplementary Figures

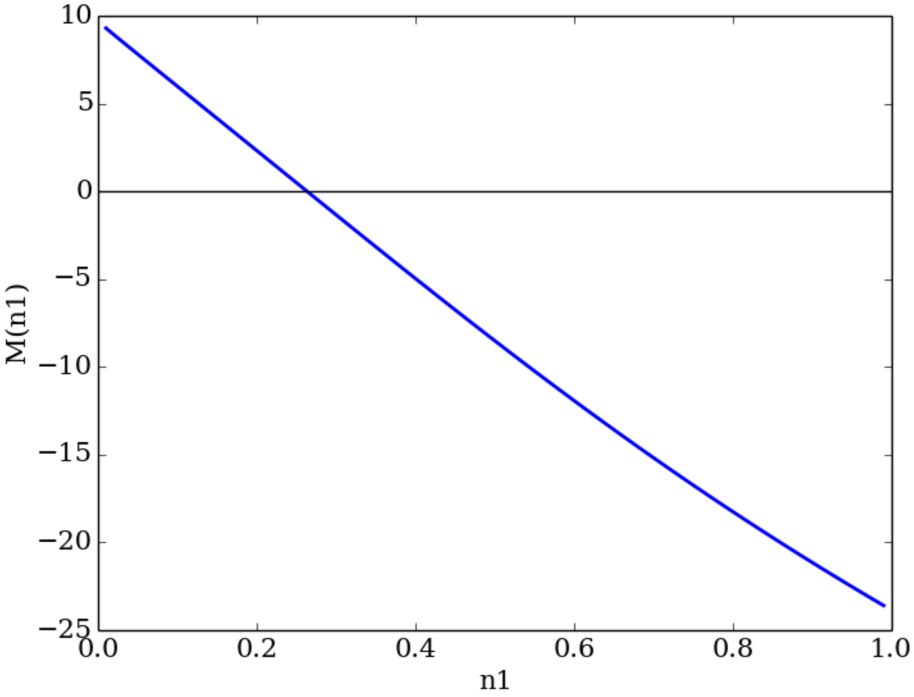

Figure S1. Unequal Frequency Analysis Plot: The plot shows ***M(n₁)*** for all values of ***n₁*** ranging from .01 to ***1***, based on the 171 DOMs obtained from MS patients. According to the plot, ***M(n₁)*** intersects with the x-axis when **n₁** is approximately .31.

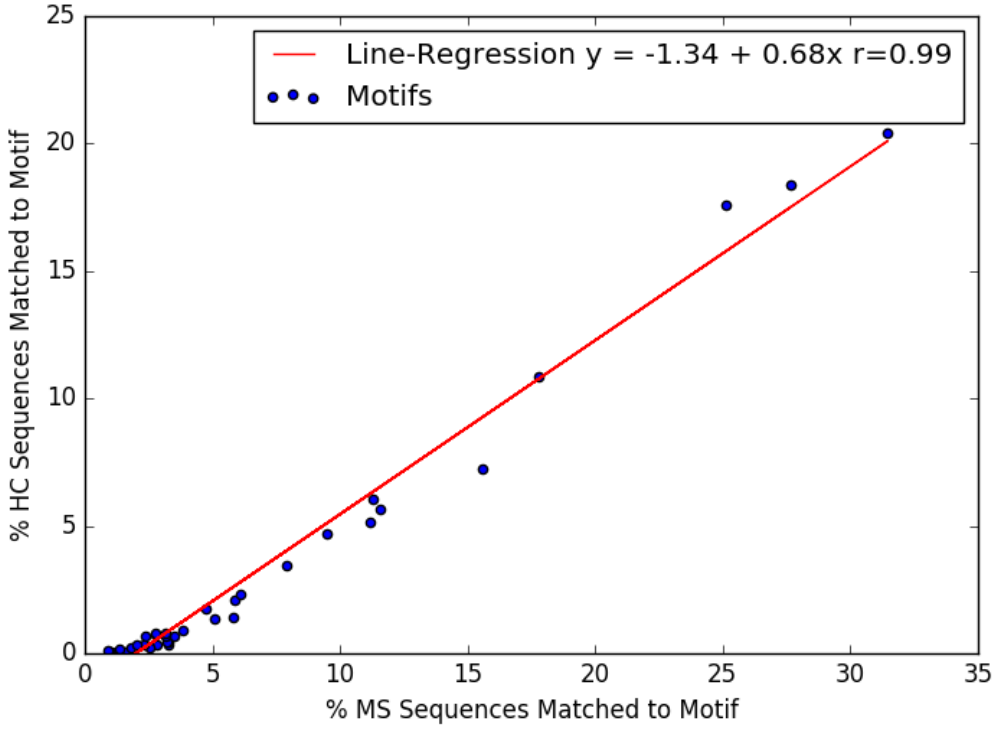

Figure S2. Percent HOMER Matches to Patient and Control Data. Each data point in the scatter plot represents a single nucleotide motif outputted by HOMER. The x-axis contains the percentage of MS sequences matched by each motif, while the y-axis contains the percentage of Healthy Control matches. The relationship between the two is strongly linear, as indicated by the regression plot where the coefficient of determination equals .99. A slope coefficient of .68 implies that the discovered HOMER motifs are not strongly over-represented in the MS data.

Supplementary Table 1

Patient ID	Total Sequence Count	Unique CDR3 Count	Diagnosis	Age	Sex	Treatment
MS-1	140270	23196	RRMS	22	M	-
MS-2	105009	11877	RRMS	22	M	IFNb
MS-3	159181	20114	RRMS	35	M	GA
MS-4	147638	16329	RRMS	55	F	GA
MS-5	144116	14350	RRMS	38	M	IFNb
MS-6	154965	28817	RRMS	38	M	-
MS-7	125948	9601	RRMS	57	F	-
MS-8	151111	19500	RRMS	48	M	IFNb
MS-9	128657	11566	CIS	42	M	-
MS-10	174883	30437	RRMS	41	M	IFNb
MS-11	100397	15568	RRMS	45	F	-
MS-12	120701	11099	SPMS	45	F	RTX
MS-13	112333	17403	RRMS	65	F	-
MS-14	86457	10780	RRMS	54	F	GA
MS-15	133502	30729	RRMS	24	M	IFNb
MS-16	110124	19232	CIS	30	F	-
MS-17	92349	12424	RRMS	41	M	GA
MS-18	91068	13973	CIS	54	F	IFNb
MS-19	115840	23758	RRMS	23	M	NTZ
MS-20	90535	20276	RRMS	20	M	-
MS-21	76310	13746	RRMS	59	M	NTZ
MS-22	85642	13656	RRMS	51	F	-
MS-23	91667	16763	RRMS	40	F	IFNb
MS-24	80830	15532	CIS	59	F	-
MS-25	82197	9351	RRMS	60	F	NTZ
MS-26	94500	20460	RRMS	33	M	GA
MS-27	123342	17567	RRMS	45	F	-
MS-28	90353	14333	RRMS	45	M	-
MS-29	67425	9335	RRMS	53	F	GA
MS-30	67250	6846	RRMS	54	F	-
MS-31	42359	6049	SPMS	58	F	-
MS-32	65648	7708	RRMS	47	F	IFNb
MS-33	88987	15253	RRMS	41	M	IFNb
MS-34	110277	20375	RRMS	36	F	-
MS-35	88528	24424	RRMS	32	M	GA
MS-36	169533	23230	RRMS	38	M	GA
MS-37	75081	17987	RRMS	36	F	IFNb

MS-38	78202	15732	RRMS	40	F	-
MS-39	69820	15962	RRMS	41	F	GA
MS-40	96619	13086	RRMS	44	F	-
MS-41	109646	19229	RRMS	33	F	IFNb
MS-42	85661	12755	RRMS	44	M	GA
MS-43	75513	8667	RRMS	45	F	-
MS-44	87332	12053	RRMS	36	F	IFNb
MS-45	86150	11867	RRMS	47	F	GA
MS-46	129040	31907	RRMS	40	M	-
MS-47	116985	21962	RRMS	43	F	GA
MS-48	75932	17793	RRMS	43	M	GA
MS-49	81947	9187	RRMS	54	F	-
MS-50	87772	16641	RRMS	41	F	-
MS-51	91379	17869	RRMS	48	M	GA
HC-1	77013	15242	HC	42	M	
HC-2	78334	17608	HC	59	F	
HC-3	67813	8373	HC	60	M	
HC-4	84824	17075	HC	30	F	
HC-5	56960	11758	HC	42	M	
HC-6	79502	16379	HC	38	M	
HC-7	68389	12063	HC	54	F	
HC-8	72647	16748	HC	62	F	
HC-9	80926	10839	HC	46	M	
HC-10	88889	16732	HC	52	M	
HC-11	103474	17984	HC	49	F	
HC-12	98008	12976	HC	50	M	
HC-13	103292	17602	HC	26	F	
HC-14	66398	11023	HC	53	M	
HC-15	108962	12027	HC	35	F	
HC-16	99223	11582	HC	27	F	
HC-17	81008	8167	HC	38	M	
HC-18	53661	3719	HC	31	M	
HC-19	107595	20033	HC	43	F	
HC-20	89043	17104	HC	24	F	
HC-21	97954	18337	HC	33	F	
HC-22	29733	10620	HC	67	F	
HC-23	62154	11076	HC	64	F	
HC-24	106375	21320	HC	27	M	
HC-25	81323	22914	HC	37	M	
HC-26	95559	25916	HC	22	M	
HC-27	97963	16721	HC	37	M	

HC-28	70407	9822	HC	22	M	
HC-29	70163	10336	HC	33	F	
HC-30	70081	12541	HC	21	M	
HC-31	89185	19278	HC	37	M	
HC-32	90114	20401	HC	32	M	
HC-33	59092	10659	HC	26	F	
HC-34	62218	7966	HC	23	F	
HC-35	108042	21800	HC	53	F	
HC-36	97131	13696	HC	58	M	
HC-37	112072	18529	HC	26	M	
HC-38	130037	19360	HC	35	F	
HC-39	125611	18959	HC	37	M	
HC-40	134607	22045	HC	35	F	
HC-41	105127	16864	HC	20	M	
HC-42	75584	13203	HC	24	M	
HC-43	81990	12488	HC	24	M	
HC-44	69458	11740	HC	34	M	
HC-45	67719	16816	HC	40	F	
HC-46	99351	14620	HC	49	M	

Sequence Counts Per Patient: The first column contains the patient id, defined as either belonging to the MS or the HC category. The second column contains the total number of database-stored sequences for each patient; some of these sequences may be identical. The third column contains the number of unique, non-redundant CDR3 sequences associated with each patient. The fourth column contains the type of MS where applicable; patients with clinically isolated syndrome (CIS) were included if MRI and cerebrospinal fluid analysis were consistent with a MS diagnosis. The fifth and sixth columns shows each patient's age and sex. The seventh columns shows the patients' therapies (IFNb, interferon beta 1; GA, glatiramer acetate; NTZ, natalizumab; RTX, rituximab); "-" indicates that the patient was not taking an approved disease-modifying therapy for MS.

Supplementary Table 2

DOM Motif	# of Patient Matches	# of Sequence Matches	%PM Overlap	%PM Non-overlap
(TNE,14),(DTA,6),(CAR,0)	35	104	100%	0%
(TNE,14),(DTA,6),(YCAR,1)	35	104	100%	0%

(TNEQ,14),(DTA,6),(CAR,0)	35	99	100%	0%
(TNEQ,14),(DTA,6),(YCAR,1)	35	99	100%	0%
(LTNE,15),(DTA,6),(CAR,0)	35	95	100%	0%
(LTNE,15),(DTA,6),(YCAR,1)	35	95	100%	0%
(TNE,14),(VYYCAR,3)	34	93	97%	3%
(TNE,14),(AVY,4),(CAR,0)	34	92	98%	2%
(TNE,14),(AVYYCAR,4)	34	92	98%	2%
(TNE,14),(DTAV,6),(CAR,0)	34	91	100%	0%
(TNE,14),(DTAV,6),(YCAR,1)	34	91	100%	0%
(TNE,14),(TAV,5),(CAR,0)	34	91	100%	0%
(TNE,14),(TAVY,5),(CAR,0)	34	91	100%	0%
(TNE,14),(TAV,5),(YCAR,1)	34	91	100%	0%
(TNE,14),(TAVYYCAR,5)	34	91	100%	0%
(TNEQ,14),(VYYCAR,3)	34	89	98%	2%
(TNEQ,14),(AVY,4),(CAR,0)	34	88	100%	0%
(TNEQ,14),(AVYYCAR,4)	34	88	100%	0%
(TNEQ,14),(DTAV,6),(CAR,0)	34	88	100%	0%
(TNEQ,14),(DTAV,6),(YCAR,1)	34	88	100%	0%
(TNEQ,14),(TAV,5),(CAR,0)	34	88	100%	0%
(TNEQ,14),(TAVY,5),(CAR,0)	34	88	100%	0%
(TNEQ,14),(TAV,5),(YCAR,1)	34	88	100%	0%
(TNEQ,14),(TAVYYCAR,5)	34	88	100%	0%
(TNEQP,14),(CAR,0)	34	87	98%	2%
(TNEQP,14),(YCAR,1)	34	87	98%	2%
(LTNE,15),(VYYCAR,3)	34	85	98%	2%
(LTNE,15),(AVY,4),(CAR,0)	34	84	100%	0%
(LTNE,15),(AVYYCAR,4)	34	84	100%	0%
(LTNE,15),(DTAV,6),(CAR,0)	34	84	100%	0%
(LTNE,15),(DTAV,6),(YCAR,1)	34	84	100%	0%
(LTNE,15),(TAV,5),(CAR,0)	34	84	100%	0%
(LTNE,15),(TAVY,5),(CAR,0)	34	84	100%	0%
(LTNE,15),(TAV,5),(YCAR,1)	34	84	100%	0%
(LTNE,15),(TAVYYCAR,5)	34	84	100%	0%
(TNEQPE,14),(CAR,0)	34	83	98%	2%
(TNEQPE,14),(YCAR,1)	34	83	98%	2%
(RDN,24),(TNE,14),(CAR,0)	34	83	98%	2%
(RDN,24),(TNE,14),(YCAR,1)	34	83	98%	2%
(SRDN,25),(TNE,14),(CAR,0)	34	83	98%	2%
(SRDN,25),(TNE,14),(YCAR,1)	34	83	98%	2%
(LTNEQP,15),(CAR,0)	34	81	98%	2%
(LTNEQP,15),(YCAR,1)	34	81	98%	2%

(RDN,24),(TNEQ,14),(CAR,0)	34	79	98%	2%
(RDN,24),(TNEQ,14),(YCAR,1)	34	79	98%	2%
(SRDN,25),(TNEQ,14),(CAR,0)	34	79	98%	2%
(SRDN,25),(TNEQ,14),(YCAR,1)	34	79	98%	2%
(LTNEQPE,15),(CAR,0)	34	78	98%	2%
(LTNEQPE,15),(YCAR,1)	34	78	98%	2%
(RDN,24),(LTNE,15),(CAR,0)	34	77	98%	2%
(RDN,24),(LTNE,15),(YCAR,1)	34	77	98%	2%
(SRDN,25),(LTNE,15),(CAR,0)	34	77	98%	2%
(SRDN,25),(LTNE,15),(YCAR,1)	34	77	98%	2%
(TNE,14),(PES,10),(CAR,0)	34	70	98%	2%
(TNE,14),(PES,10),(YCAR,1)	34	70	98%	2%
(TNEQPES,14),(CAR,0)	34	68	98%	2%
(TNEQPES,14),(YCAR,1)	34	68	98%	2%
(LTNE,15),(PES,10),(CAR,0)	34	67	98%	2%
(LTNE,15),(PES,10),(YCAR,1)	34	67	98%	2%
(LTNEQPES,15),(CAR,0)	34	66	98%	2%
(LTNEQPES,15),(YCAR,1)	34	66	98%	2%
(SKN,21),(YLT,16),(PES,10)	33	62	37%	63%
(YLTN,16),(DTA,6),(CAR,0)	32	87	94%	6%
(YLTN,16),(DTA,6),(YCAR,1)	32	87	94%	6%
(KNT,20),(YLT,16),(EQP,12)	32	69	37%	63%
(SKN,21),(YLT,16),(EQP,12)	32	62	40%	60%
(KNT,20),(YLT,16),(PES,10)	32	62	38%	62%
(SKN,21),(YLT,16),(EQPE,12)	32	58	41%	59%
(YLTN,16),(VYYCAR,3)	31	79	91%	9%
(YLTN,16),(AVY,4),(CAR,0)	31	77	93%	7%
(YLTN,16),(AVYYCAR,4)	31	77	93%	7%
(YLTN,16),(DTAV,6),(CAR,0)	31	77	93%	7%
(YLTN,16),(DTAV,6),(YCAR,1)	31	77	93%	7%
(YLTN,16),(TAV,5),(CAR,0)	31	77	93%	7%
(YLTN,16),(TAVY,5),(CAR,0)	31	77	93%	7%
(YLTN,16),(TAV,5),(YCAR,1)	31	77	93%	7%
(YLTN,16),(TAVYYCAR,5)	31	77	93%	7%
(LYLTN,17),(CAR,0)	31	76	93%	7%
(LYLTN,17),(YCAR,1)	31	76	93%	7%
(TLYLT,18),(EQP,12)	31	71	36%	64%
(KNT,20),(TNEQP,14)	31	71	39%	61%
(YLT,16),(QPE,11),(CAR,0)	31	71	91%	9%
(YLT,16),(QPE,11),(YCAR,1)	31	71	91%	9%
(KNT,20),(LTNEQP,15)	31	70	38%	62%

(NTL,19),(TNEQP,14)	31	69	39%	61%
(NTL,19),(LTNEQP,15)	31	68	38%	62%
(NTLYLT,19),(EQP,12)	31	68	36%	64%
(YLTN,16),(QPE,11),(CAR,0)	31	68	95%	5%
(YLTN,16),(QPE,11),(YCAR,1)	31	68	95%	5%
(KNT,20),(YLTNEQP,16)	31	67	38%	62%
(SKN,21),(TNEQP,14)	31	65	43%	57%
(SKN,21),(LTNEQP,15)	31	64	42%	58%
(SKN,21),(TNEQPE,14)	31	60	45%	55%
(SKNT,21),(YLT,16),(EQP,12)	31	60	41%	59%
(SKN,21),(YLTNEQP,16)	31	60	41%	59%
(SKN,21),(YLT,16),(QPES,11)	31	60	38%	62%
(SKN,21),(LTNEQPE,15)	31	59	44%	56%
(KNT,20),(YLT,16),(EQPE,12)	31	59	42%	58%
(SKNT,21),(YLT,16),(PES,10)	31	59	38%	62%
(SKN,21),(YLTN,16),(PES,10)	31	58	39%	61%
(SKN,21),(LYLT,17),(PES,10)	31	57	36%	64%
(SKN,21),(TNE,14),(PES,10)	31	57	45%	55%
(SKN,21),(LTNE,15),(PES,10)	31	56	44%	56%
(SKNT,21),(YLT,16),(EQPE,12)	31	56	42%	58%
(SKN,21),(YLTNEQPE,16)	31	56	42%	58%
(RDN,24),(YLTN,16),(CAR,0)	30	74	91%	9%
(RDN,24),(YLTN,16),(YCAR,1)	30	74	91%	9%
(SRDN,25),(YLTN,16),(CAR,0)	30	74	91%	9%
(SRDN,25),(YLTN,16),(YCAR,1)	30	74	91%	9%
(LYLTNE,17),(YCAR,1)	30	72	98%	2%
(LYLTNE,17),(CAR,0)	30	72	98%	2%
(YLT,16),(EQP,12),(CAR,0)	30	72	94%	6%
(YLT,16),(EQP,12),(YCAR,1)	30	72	94%	6%
(TLY,18),(TNEQP,14)	30	70	38%	62%
(TLYLTNEQP,18)	30	69	37%	63%
(LYLTNEQ,17),(CAR,0)	30	69	98%	2%
(LYLTNEQ,17),(YCAR,1)	30	69	98%	2%
(YLT,16),(EQPE,12),(CAR,0)	30	69	94%	6%
(YLT,16),(EQPE,12),(YCAR,1)	30	69	94%	6%
(YLTNEQP,16),(CAR,0)	30	69	98%	2%
(YLTNEQP,16),(YCAR,1)	30	69	98%	2%
(NTLY,19),(TNEQP,14)	30	67	38%	62%
(NTLYLTNEQP,19)	30	66	37%	63%
(LYLT,17),(QPE,11),(CAR,0)	30	66	90%	10%
(LYLT,17),(QPE,11),(YCAR,1)	30	66	90%	10%

(YLTNEQPE,16),(CAR,0)	30	66	98%	2%
(YLTNEQPE,16),(YCAR,1)	30	66	98%	2%
(YLT,16),(PES,10),(CAR,0)	30	65	86%	14%
(YLT,16),(PES,10),(YCAR,1)	30	65	86%	14%
(TLYLT,18),(PES,10)	30	64	39%	61%
(TLYLT,18),(QPE,11)	30	64	39%	61%
(SKNT,21),(TNEQP,14)	30	62	43%	57%
(SKNT,21),(LTNEQP,15)	30	61	42%	58%
(NTLYLT,19),(PES,10)	30	61	39%	61%
(NTLYLT,19),(QPE,11)	30	61	39%	61%
(YLT,16),(QPES,11),(CAR,0)	30	61	90%	10%
(YLT,16),(QPES,11),(YCAR,1)	30	61	90%	10%
(TLYLT,18),(EQPE,12)	30	60	41%	59%
(KNT,20),(TNEQPE,14)	30	60	45%	55%
(KNT,20),(YLT,16),(QPES,11)	30	60	40%	60%
(YLTN,16),(PES,10),(CAR,0)	30	60	93%	7%
(YLTN,16),(PES,10),(YCAR,1)	30	60	93%	7%
(KNT,20),(LTNEQPE,15)	30	59	44%	56%
(KNT,20),(TNE,14),(YCA,1)	30	59	59%	41%
(KNT,20),(LTNE,15),(YCA,1)	30	58	58%	42%
(KNT,20),(TNE,14),(YYCA,2)	30	58	58%	42%
(NTL,19),(TNEQPE,14)	30	58	44%	56%
(KNT,20),(TNEQ,14),(YCA,1)	30	58	60%	40%
(TLY,18),(TNE,14),(YCA,1)	30	58	56%	44%
(SKNT,21),(YLTNEQP,16)	30	58	43%	57%
(KNT,20),(YLTN,16),(PES,10)	30	58	41%	59%
(YLTN,16),(QPES,11),(CAR,0)	30	58	94%	6%
(SKN,21),(YLTN,16),(QPES,11)	30	58	39%	61%
(YLTN,16),(QPES,11),(YCAR,1)	30	58	94%	6%
(SKNT,21),(YLT,16),(QPES,11)	30	58	39%	61%
(KNT,20),(LTNE,15),(YYCA,2)	30	57	57%	43%
(NTL,19),(LTNEQPE,15)	30	57	43%	57%
(TLYLTNE,18),(YCA,1)	30	57	56%	44%
(NTLYLT,19),(EQPE,12)	30	57	42%	58%
(KNT,20),(TNEQ,14),(YYCA,2)	30	57	59%	41%
(SKNT,21),(TNEQPE,14)	30	57	45%	55%
(TLY,18),(TNE,14),(YYCA,2)	30	57	56%	44%
(KNT,20),(YLTNEQPE,16)	30	57	43%	57%
(KNTLYLT,20),(PES,10)	30	56	39%	61%
(SKNT,21),(LTNEQPE,15)	30	56	44%	56%
(TLYLTNE,18),(YYCA,2)	30	56	55%	45%

(KNT,20),(TNE,14),(PES,10)	30	56	46%	54%
(SKN,21),(TNEQPES,14)	30	56	46%	54%
(KNT,20),(LTNE,15),(PES,10)	30	55	45%	55%
(SKN,21),(LTNEQPES,15)	30	55	45%	55%
(SKNT,21),(YLTNEQPE,16)	30	54	44%	56%

Discovered DOM Motifs: All 171 discovered DOMs are listed in this table. The first column represents each DOM in a parenthesis notation where *(S,x)* represents an non-gapped subsequence *S* that is located *x* amino acids to the left of the CDR3. The second column contains the total number of MS patient matches to the DOM. The third columns the total number of sequence matches to the DOM. The fourth column illustrates the percentage of matching sequences that also match to the master motif. The fifth column illustrates the percentage of matching sequences that do not match to the master motif. The Prime Motif, which matches the maximum of sequences and the maximum number of patients, appears in the first row of the table.

Supplementary Table 3

Patient ID	V-Gene	# Sequence Matches
MS-5	IGHV3-48	10
MS-15	IGHV3-7	9
MS-20	IGHV3-48	7
MS-11	IGHV3-7	5
MS-13	IGHV3-30/33rn	5
MS-26	IGHV3-74	5
MS-8	IGHV3-23	4
MS-19	IGHV3-30/33rn	4
MS-27	IGHV3-7	4
MS-28	IGHV3-53/66	4
MS-1	IGHV3-72	3
MS-2	IGHV3-11	3
MS-6	IGHV3-30/33rn	3
MS-12	IGHV3-21	3
MS-17	IGHV3-21	3
MS-23	IGHV3-53/66	3
MS-25	IGHV3-72	3
MS-3	IGHV3-11	2
MS-9	IGHV3-21	2

MS-10	IGHV3-53/66	2
MS-14	IGHV3-30/33rn	2
MS-16	IGHV3-74	2
MS-18	IGHV3-21	2
MS-30	IGHV3-30/33rn	2
MS-31	IGHV3-30/33rn	2
MS-4	IGHV3-21	1
MS-7	IGHV3-53/66	1
MS-21	IGHV3-7	1
MS-22	IGHV3-30/33rn	1
MS-24	IGHV3-48	1
MS-32	IGHV3-7	1
MS-40	IGHV3-30/33rn	1
MS-42	IGHV3-48	1
MS-44	IGHV3-53/66	1
MS-46	IGHV3-74	1

Prime Motif Patient Matches: All 104 sequence matches to the Prime Motif are grouped by patient in this table. The first column lists the ids of the patients with matching sequences to the prime motif. The second column lists the most frequently occurring v-gene among the matching sequences. The final column contains the total number of matching sequences associated with an individual patient. A total of 35 patients are present in the table. All matching v-genes are IGHV3.

Supplementary Table 4

Patient ID	V-Gene	# Sequence Matches
MS-15	IGHV3-23	14
MS-5	IGHV3-48	13
MS-20	IGHV3-48	12
MS-12	IGHV3-23	11
MS-11	IGHV3-7	10
MS-14	IGHV3-48	10
MS-1	IGHV3-7	9
MS-8	IGHV3-23	9
MS-23	IGHV3-53/66	9
MS-18	IGHV3-30/33rn	7
MS-24	IGHV3-23	7

MS-26	IGHV3-30/33rn	7
MS-3	IGHV3-11	6
MS-9	IGHV3-23	6
MS-13	IGHV3-30/33rn	6
MS-19	IGHV3-23	6
MS-25	IGHV3-72	6
MS-17	IGHV3-21	5
MS-21	IGHV3-53/66	5
MS-27	IGHV3-7	5
MS-28	IGHV3-53/66	5
MS-2	IGHV3-11	4
MS-6	IGHV3-30/33rn	4
MS-10	IGHV3-7	4
MS-22	IGHV3-30/33rn	4
MS-7	IGHV3-53/66	3
MS-16	IGHV3-74	3
MS-30	IGHV3-30/33rn	3
MS-31	IGHV3-30/33rn	3
MS-4	IGHV3-21	2
MS-29	IGHV3-23	2
MS-32	IGHV3-7	2
HC-10	IGHV3-15	2
HC-4	IGHV3-72	1
MS-38	IGHV3-23	1
MS-40	IGHV3-30/33rn	1
MS-42	IGHV3-48	1
MS-44	IGHV3-53/66	1
MS-46	IGHV3-74	1
MS-49	IGHV3-23	1
MS-50	IGHV3-73	1
HC-15	IGHV3-23	1
HC-20	IGHV3-64	1
HC-24	IGHV3-7	1
HC-29	IGHV3-7	1
HC-42	IGHV3-7	1

Selective Component Patient Matches: 46 individuals match the selective components TNE and DTA. 11 of these individuals do not match the Prime Motif. Each row in the table represents a patient match to the selective components, as indicated by the patient id in the first column. A total of 7 Healthy Control matches are marked in green. The four MS patients that do not contain the Prime Motif are marked in orange. The count of selective component sequence matches per patient is listed in column three. These sequence counts range from values of one to fourteen. The second column lists the most frequently occurring v-gene among the matching sequences. All matching sequences contain the IGHV3 variable region.

Supplementary Table 5

Patient ID	%IGHV3 Usage
HC-34	62.06%
HC-41	57.05%
HC-29	54.92%
HC-42	52.81%
HC-23	52.29%
MS-42	51.88%
MS-34	51.63%
HC-46	51.46%
MS-39	51.41%
HC-21	51.31%
MS-23	50.44%
HC-13	50.11%
HC-30	50.08%
MS-7	50.07%
MS-16	49.48%
HC-37	49.35%
HC-5	49.24%
MS-41	49.17%
MS-25	48.90%
HC-36	48.65%
HC-44	48.51%
HC-35	48.49%
HC-40	48.42%
HC-19	48.41%
HC-28	48.30%

HC-27	48.03%
HC-38	47.92%
MS-22	47.74%
MS-20	47.74%
MS-37	47.53%
MS-33	47.29%
HC-22	47.13%
MS-46	47.07%
HC-10	46.88%
MS-13	46.80%
HC-26	46.80%
HC-8	46.61%
HC-14	46.49%
MS-1	46.39%
HC-31	46.36%
MS-35	46.19%
HC-17	46.08%
MS-11	46.00%
MS-29	45.97%
HC-3	45.88%
MS-12	45.86%
MS-5	45.61%
MS-26	45.57%
MS-47	45.54%
MS-38	45.43%
HC-24	45.38%
MS-19	45.36%
HC-16	45.32%
HC-39	45.30%
MS-48	44.88%
MS-8	44.18%
MS-14	43.96%
HC-1	43.90%
HC-2	43.41%
HC-32	43.20%
MS-50	43.20%
HC-43	42.99%
HC-12	42.93%
MS-15	42.92%
MS-40	42.78%
HC-18	42.69%

HC-6	42.52%
HC-20	42.31%
MS-49	42.17%
HC-4	42.01%
HC-11	41.61%
MS-4	41.57%
MS-10	41.49%
MS-18	41.42%
HC-25	41.42%
MS-30	41.29%
HC-15	40.90%
MS-2	40.40%
HC-33	40.29%
MS-21	40.23%
MS-44	40.16%
MS-31	40.05%
MS-43	40.03%
MS-27	39.85%
MS-45	39.73%
MS-32	38.73%
HC-9	38.65%
HC-7	38.49%
MS-17	38.33%
MS-51	38.14%
HC-45	36.89%
MS-3	36.73%
MS-36	36.45%
MS-9	35.09%
MS-24	34.65%
MS-6	34.54%
MS-28	33.95%

IGHV3 Percent Usage Per Patient: Column one contains the patient id. Column two contains the percentage of non-redundant sequences within each patient that may be categorized as IGHV3. Non-redundancy is defined by the presence of a unique CDR3. The table is sorted by the v-gene percentage in column two. Certain rows within the table are colored by match type. MS patients matching to the Prime motif are colored in blue. Healthy Controls matching to the selective components TNE and DTA are colored green. MS patients not matching the Prime Motif but matching TNE and DTA are colored in orange. All remaining rows are colored white. The color distribution in the table does not appear related to v-gene usage.

Supplementary Table 6

Patient ID	# Sequence Matches
MS-5	10
MS-15	9
MS-20	7
MS-26	5
MS-13	5
MS-27	4
MS-28	4
MS-8	4
MS-11	4
MS-23	3
MS-6	3
MS-2	3
MS-1	3
MS-12	3
MS-17	3
MS-19	3
MS-25	2
MS-3	2
MS-9	2
MS-10	2
MS-16	2
MS-14	2
MS-18	2
MS-31	2
MS-30	2
MS-40	1
MS-42	1
MS-44	1
MS-46	1
MS-32	1
MS-24	1
MS-22	1
MS-21	1
MS-7	1
MS-4	1

Indel Requirment Prime Motif Patient Matches: All 101 sequences that comply with the indel requirement have been grouped by patient in this table. Sequence counts that differ from the original Prime Motif matching counterparts have highlighted in yellow. Three Prime Motif matching sequences from three different patients do not comply with the indel requirement.

Supplementary Table 7

Patient ID	Run ID	Lane ID	Primer Set 1	Primer Set 2	Motif Detected?
MS-1	3	2		IgG29	YES
MS-1	4	1	IgM83	IgG29	YES
MS-1	4	2	IgM83	IgG29	YES
MS-1	4	2	IgM83	IgG29	YES
MS-1	4	2	IgM83	IgG29	YES
MS-1	6	1	IgM83		YES
MS-2	1	1	IgM83	IgG29	YES
MS-2	1	2	IgM83	IgG29	YES
MS-3	1	1	IgM83	IgG29	YES
MS-3	1	2	IgM83	IgG29	YES
MS-4	1	1	IgM83	IgG29	YES
MS-4	1	2	IgM83	IgG29	YES
MS-5	1	1	IgM83	IgG29	YES
MS-5	1	2	IgM83	IgG29	YES
MS-6	1	1	IgM83	IgG29	YES
MS-6	1	2	IgM83	IgG29	YES
MS-7	4	1	IgM83	IgG29	YES
MS-7	4	2	IgM83	IgG29	YES
MS-8	1	1	IgM83	IgG29	YES
MS-8	1	2	IgM83	IgG29	YES
MS-9	1	1	IgM83	IgG29	YES
MS-9	1	2	IgM83	IgG29	YES
MS-10	1	1	IgM83	IgG29	YES
MS-10	1	2	IgM83	IgG29	YES
MS-11	2	1	IgM83	IgG29	YES
MS-11	2	2	IgM83	IgG29	YES
MS-11	5	1		IgG29	YES
MS-12	4	1	IgM83	IgG29	YES
MS-12	4	2	IgM83	IgG29	YES
MS-13	4	1	IgM83	IgG29	YES
MS-13	4	2	IgM83	IgG29	YES

MS-14	2	1	IgM83	IgG29	YES
MS-14	2	2	IgM83	IgG29	YES
MS-14	5	1		IgG29	YES
MS-15	2	1	IgM83	IgG29	YES
MS-15	2	2	IgM83	IgG29	YES
MS-15	5	1		IgG29	YES
MS-16	2	1	IgM83	IgG29	YES
MS-16	2	2	IgM83	IgG29	YES
MS-16	5	1		IgG29	YES
MS-17	2	1	IgM83	IgG29	YES
MS-17	2	2	IgM83	IgG29	YES
MS-17	5	1		IgG29	YES
MS-18	2	1	IgM83	IgG29	YES
MS-18	2	2	IgM83	IgG29	YES
MS-18	5	1		IgG29	YES
MS-19	4	1	IgM83	IgG29	YES
MS-19	4	2	IgM83	IgG29	YES
MS-20	2	1	IgM83	IgG29	YES
MS-20	2	2	IgM83	IgG29	YES
MS-20	5	1		IgG29	YES
MS-21	2	1	IgM83	IgG29	YES
MS-21	2	2	IgM83	IgG29	YES
MS-21	5	1		IgG29	YES
MS-22	3	1	IgM83	IgG29	YES
MS-22	3	2	IgM83	IgG29	YES
MS-22	5	1		IgG29	YES
MS-23	3	1	IgM83	IgG29	YES
MS-23	3	2	IgM83	IgG29	YES
MS-23	5	1		IgG29	YES
MS-24	3	1	IgM83	IgG29	YES
MS-24	3	2	IgM83	IgG29	YES
MS-24	5	1		IgG29	YES
MS-25	4	1	IgM83	IgG29	YES
MS-25	4	2	IgM83	IgG29	YES
MS-26	3	1	IgM83	IgG29	YES
MS-26	3	2	IgM83	IgG29	YES
MS-26	5	1		IgG29	YES
MS-27	4	1	IgM83	IgG29	YES
MS-27	4	2	IgM83	IgG29	YES
MS-28	3	1	IgM83	IgG29	YES
MS-28	3	2	IgM83	IgG29	YES
MS-28	5	1		IgG29	YES

MS-29	3	1	IgM83	IgG29	NO
MS-29	3	2	IgM83	IgG29	NO
MS-29	5	1		IgG29	NO
MS-30	4	1	IgM83	IgG29	YES
MS-30	4	2	IgM83	IgG29	YES
MS-31	3	1	IgM83	IgG29	YES
MS-31	3	2	IgM83	IgG29	YES
MS-31	5	1		IgG29	YES
MS-32	3	1	IgM83	IgG29	YES
MS-32	3	2	IgM83	IgG29	YES
MS-32	5	1		IgG29	YES
MS-33	6	1	IgM83	IgG29	NO
MS-33	6	2	IgM83	IgG29	NO
MS-34	6	1	IgM83	IgG29	NO
MS-34	6	2	IgM83	IgG29	NO
MS-35	7	1	IgM83	IgG29	NO
MS-35	7	2	IgM83	IgG29	NO
MS-36	9	1	IgM83	IgG29	NO
MS-36	9	2	IgM83	IgG29	NO
MS-37	7	1	IgM83	IgG29	NO
MS-37	7	2	IgM83	IgG29	NO
MS-38	7	1	IgM83	IgG29	NO
MS-38	7	2	IgM83	IgG29	NO
MS-39	7	1	IgM83	IgG29	NO
MS-39	7	2	IgM83	IgG29	NO
MS-40	9	1	IgM83	IgG29	YES
MS-40	9	2	IgM83	IgG29	YES
MS-41	7	1	IgM83	IgG29	NO
MS-41	7	2	IgM83	IgG29	NO
MS-42	7	1	IgM83	IgG29	YES
MS-42	7	2	IgM83	IgG29	YES
MS-43	9	1	IgM83	IgG29	NO
MS-43	9	2	IgM83	IgG29	NO
MS-44	8	1	IgM83	IgG29	YES
MS-44	8	2	IgM83	IgG29	YES
MS-45	8	1	IgM83	IgG29	NO
MS-45	8	2	IgM83	IgG29	NO
MS-46	9	1	IgM83	IgG29	YES
MS-46	9	2	IgM83	IgG29	YES
MS-47	8	1	IgM83	IgG29	NO
MS-47	8	2	IgM83	IgG29	NO
MS-48	8	1	IgM83	IgG29	NO

MS-48	8	2	IgM83	IgG29	NO
MS-49	9	1	IgM83	IgG29	NO
MS-49	9	2	IgM83	IgG29	NO
MS-50	9	1	IgM83	IgG29	NO
MS-50	9	2	IgM83	IgG29	NO
MS-51	9	1	IgM83	IgG29	NO
MS-51	9	2	IgM83	IgG29	NO

MS Patient Batch Runs: This table contains the separate sequencing runs and samples for all the MS patients, along with whether the Prime Motif had been identified in a particular patient or not. Based on the table, every MS patient that did not contain the Prime Motif shared its run-lane combinations with at-least one patient in whom the motif was present.
